# Polyaromatic Hydrocarbon (PAH)-Based *Aza*-POPOPs: Synthesis, Photophysical Studies, and Nitroanalyte Sensing Abilities

**DOI:** 10.3390/ijms241210084

**Published:** 2023-06-13

**Authors:** Mohammed S. Mohammed, Igor S. Kovalev, Natalya V. Slovesnova, Leila K. Sadieva, Vadim A. Platonov, Alexander S. Novikov, Sougata Santra, Julia E. Morozova, Grigory V. Zyryanov, Valery N. Charushin, Brindaban C. Ranu

**Affiliations:** 1 Department of Organic and Biomolecular Chemistry, Ural Federal University, 19, Mira St., 620002 Yekaterinburg, Russia; samirmohammed422@gmail.com (M.S.M.); ekls85@yandex.ru (I.S.K.); saarge@mail.ru (N.V.S.); l.k.sadieva@urfu.ru (L.K.S.); vplatonov10@mail.ru (V.A.P.); sougatasantra85@gmail.com (S.S.); gvzyryanov@gmail.com (G.V.Z.); charushin@ios.uran.ru (V.N.C.); bcranu@gmail.com (B.C.R.); 2 Department of Pharmacy and Chemistry, Ural Medical University, 3, Repina St., 620028 Yekaterinburg, Russia; 3 I. Ya. Postovsky Institute of Organic Synthesis of RAS (Ural Division), 22/20, S. Kovalevskoy/Akademicheskaya St., 620137 Yekaterinburg, Russia; 4 Institute of Chemistry, Saint Petersburg State University, Universitetskaya Nab., 7/9, 199034 Saint Petersburg, Russia; a.s.novikov@spbu.ru; 5 Research Institute of Chemistry, Peoples’ Friendship University of Russia (RUDN University), Miklukho-Maklaya St., 6, 117198 Moscow, Russia; 6 Arbuzov Institute of Organic and Physical Chemistry, FRC Kazan Scientific Center of RAS, Arbuzov Str. 8, 420088 Kazan, Russia; 7 School of Chemical Sciences, Indian Association for the Cultivation of Science, Jadavpur, Kolkata 700032, India

**Keywords:** *aza*-POPOPs, 1,4-bis(5-phenyl-2-oxazolyl)benzene analogues, click reactions, photophysical studies, fluorescence quenching, nitro-explosives, chemosensors

## Abstract

1,4-Bis(5-phenyl-2-oxazolyl)benzene (POPOP) is a common scintillation fluorescent laser dye. In this manuscript, the synthesis of 2-Ar-5-(4-(4-Ar’-1*H*-1,2,3-triazol-1-yl)phenyl)-1,3,4-oxadiazoles (Ar, Ar’ = Ph, naphtalenyl-2, pyrenyl-1, triphenilenyl-2), as PAH-based *aza*-analogues of POPOP, by means of Cu-catalyzed click reaction between 2-(4-azidophenyl)-5-Ar-1,3,4-oxadiazole and terminal ethynyl-substituted PAHs is reported. An investigation of the photophysical properties of the obtained products was carried out, and their sensory response to nitroanalytes was evaluated. In the case of pyrenyl-1-substituted *aza*-POPOP, dramatic fluorescence quenching by nitroanalytes was observed.

## 1. Introduction

**Figure 1:**
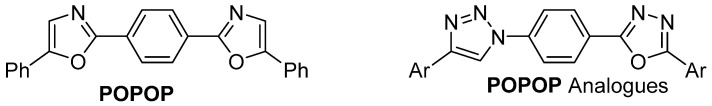
POPOP and its *aza*-analog core. POPOP = 1,4-Bis(5-phenyl-2-oxazolyl)benzene.

One of the most important tasks of modern synthetic organic chemistry is obtaining new compounds that will find wide application in various industrial areas and medicine. One of the most representative examples of a novel type of compounds is azaheterocyclic fluorophores, which can also act as drug candidates and contain cyclic azole or azine core as a common pharmacophore or ligand unit, as well as fused or conjugated (poly)aromatic moieties as fluorogenes and/or receptor units. 1,4-Bis(5-phenyl-2-oxazolyl)benzene (POPOP) is a well-known organic fluorophore (Figure 1), which is commonly used as a spectrum shifter, including in multilayer scintillation screen to visualize radiation that is invisible to the human eye [1,2]. Due to its excellent photophysical properties, especially its high quantum yield (up to 97.5% in cyclohexane or 91% in ethanol [3]), POPOP is successfully utilized in dye vapor lasers [4,5]. On the other hand, *aza*-analogues of POPOP, such as 1,3,4-oxadiazoles, are of wide interest due to the promising biological activity of oxadizoles [6,7,8] and their intriguing photophysical properties [9,10,11,12].

The term “click chemistry” was introduced for the first time in 1998 by K. Barry Sharpless, the 2001 and 2022 Nobel Prize Laureate in Chemistry, and it was fully described by B.K. Sharpless, H. C. Kolb, and M.G. Finn in 2001 [13]. Since then, this approach has gained worldwide acknowledgment for its simple reaction technique leading to single product formation without by-products. The copper(I)-catalyzed azide/alkyne “click” reaction (also termed Sharpless “click” reaction) occurs through the interaction between a terminal alkyne and an azide in the presence of Cu(I) catalysis, and results in a cycloaddition product—1,4 disubstituted 1,2,3-triazole [14,15,16,17]. This reaction is also known as Cu(I)-catalyzed Huisgen 1,3-dipolar cycloaddition between alkynes and azides (CuAAC) [18,19]. Additionally, 1,3-dipolar cycloaddition of organic azides with alkynes as dipolarophiles is the most straightforward way to obtain useful 1,2,3-triazoles [5]. Applications of the Cu-catalyzed (cyclo)addition reactions have already contributed to many areas of modern chemistry, including asymmetric synthesis [20,21,22,23,24], and Cu(I)-catalyzed azide–alkyne cycloaddition (CuAAC) is a convenient tool for bioconjugation reactions, peptidomimetic chemistry, polymer and materials sciences, and supramolecular chemistry [25,26]. The chemistry of 1,2,3-triazoles has gained much attention since its discovery, and various synthetic protocols have been developed for the synthesis of this moiety [27,28]. In addition, 1,2,3-triazoles are one of the most important connective linkers and functional aromatic heterocycles in modern chemistry [29]. In addition, it is well known that 1,2,3-triazoles, as highly valuable *N*-heterocyclic compounds, are ubiquitous in many pharmaceuticals and bioactive molecules [30,31].

In this manuscript, we report the synthesis of novel POPOP *aza*-analogues (Figure 1) via the click reaction between 2-(4-azidophenyl)-5-(aryl)-oxadiazole-1,3,4 and ethynyl-substituted (poly)arenes, as well as an investigation of the photophysical and sensing properties of the obtained products.

## 2. Results

### 2.1. Synthesis of Target Fluorophores

**Scheme 1:**
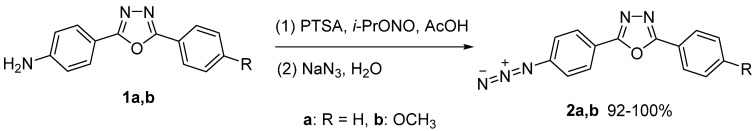
Synthesis of the azido components **2a**,**b**. PTSA = *p*-Toluenesulfonic acid.

As one of the azole fragments, the *aza*-analog of 1,3-oxazole was used as it is easily derived synthetically [32,33] from 1,3,4-oxadiazol. As mentioned above, 1,3,4-oxadiazole fragment is widely presented in many compounds with various promising biological activities and possesses intriguing photophysical properties. 1,2,3-triazole, an azadeoxa analogue of 1,3-oxazole, was introduced as second azole by using Cu(I)-catalyzed click reaction. Based on these click reactions, we successfully synthesized 4-azidophenyloxadiazoles **2a**,**b** as precursors of the azido components. These 4-azidophenyloxadiazoles were prepared for the first time by means of modified Sandmeyer reaction starting from 4-(5-phenyl-1,3,4-oxadiazol-2-yl)anilines [34] (Scheme 1).

**Scheme 2:**
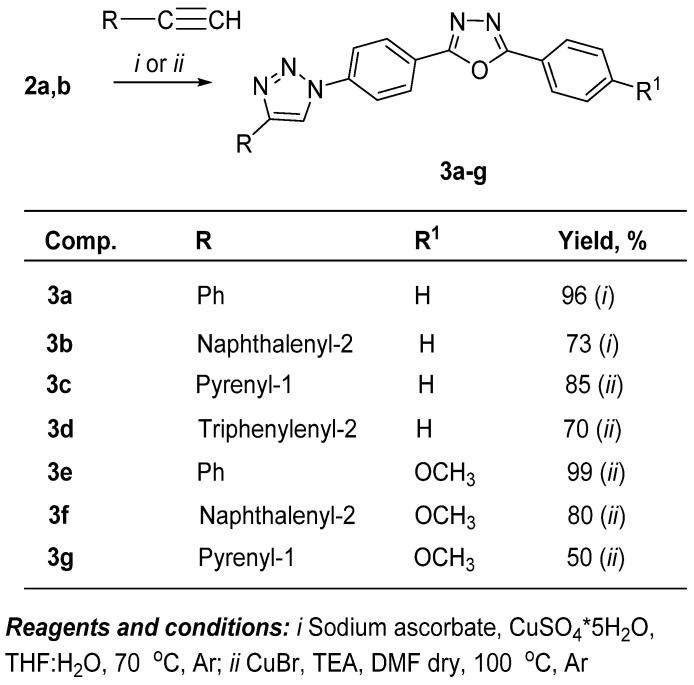
Synthesis of the POPOP analogues **3**.

As a second step, we used Cu(I)-promoted azido–alkyne coupling (CuAAC) (Scheme 2) to construct the 1,2,3-triazole ring by using two different approaches. The first approach involved the use of cuprous sulphate derived in situ via the reaction of sodium ascorbate with copper(II) sulphate pentahydrate in aqueous THF (H_2_O:THF 1:9 *v*/*v*) to obtain the target products **3** in 73–96% yields. The second approach involved modified reaction conditions, namely using cuprous bromide in dry DMF. The main reason for using dry DMF is the poor solubility of the starting PAH-ethynyl derivatives in aqueous THF. The target products **3** were obtained in 50–99% yields. It is worth mentioning that the main advantage of the reported copper(I)-catalized azido–alkyne coupling over the classical Huisgen 1,3-dipolar cycloaddition [35] is that the high regioselectivity of the former reaction results in only 1,4-isomer, whereas non-catalyzed 1,3-dipolar cycloaddition gives a mixture of 1,4- and 1,5-isomers [36].

The structures of all the obtained compounds were confirmed by means of ^1^H and ^13^C NMR spectroscopy, mass spectrometry, and elemental analysis (See ESI for details).

### 2.2. Photophysical and Sensing Properties of the Obtained Compounds

The above-mentioned azaheterocycles **3** could be considered as POPOP aza-analogs. However, initial photophysical studies showed that the photophysical properties of the obtained compounds **3** are not quite similar to those of POPOP, which are, probably, due to the contribution of both peripheral (poly)aromatic substituents and azole moieties. Thus, in the spectra of pyrene-bearing fluorophores **3c** and **3g**, both absorption and emission are red-shifted in comparison with POPOP, while the emission spectra of other fluorophores are blue-shifted compared to that of POPOP. Among all the fluorophores, a pronounced blue shift is observed for fluorophore **3a**. Additionally, the emission spectrum of **3a** is the most blue-shifted in comparison to the emission spectra of all other POPOP analogues. Whereas the absorption and emission spectra of POPOP have a weakly expressed vibronic structure, the spectra of all fluorophores **3**, including pyrene-containing ones, are blurred. Interestingly, while S_0_→S_1_ electronic transition has a higher intensity than S_0_→S_2_ transition in POPOP and its analogues **3c** and **3e**, it is the other way around in the other analogues.

**Figure 2:**
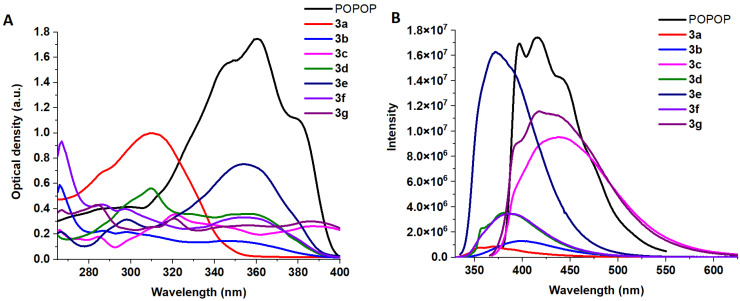
Absorption (**A**) and emission (**B**) spectra of POPOP and its *aza*-analogues **3** in CH_2_Cl_2_ (10^−5^ M). POPOP = 1,4-Bis(5-phenyl-2-oxazolyl)benzene.

The quantum yield values of the obtained fluorophores vary from 17 to 98%. The introduction of a methoxy group into the aromatic substituent in the 1,3,4-oxazole core results in an increase in the quantum yield. It is especially clearly observed in the values of the quantum yields of pyrenyl-containing analogues **3c** (23%) and **3g** (84%)—the introduction of a methoxy group results in a dramatical increase in their quantum yields. (Figure 2, Table 1).

In addition, the introduction of a pyrene moiety results in an increase in the fluorescence lifetime values to 4.52 ns (**3c**) and 4.34 ns (**3g**), which are similar to the data reported for other pyrene derivatives in the literature [37,38,39]. For the other fluorophores **3**, the lifetime values vary from 0.49 to 1.22 ns (Table 1).

**Figure 3:**
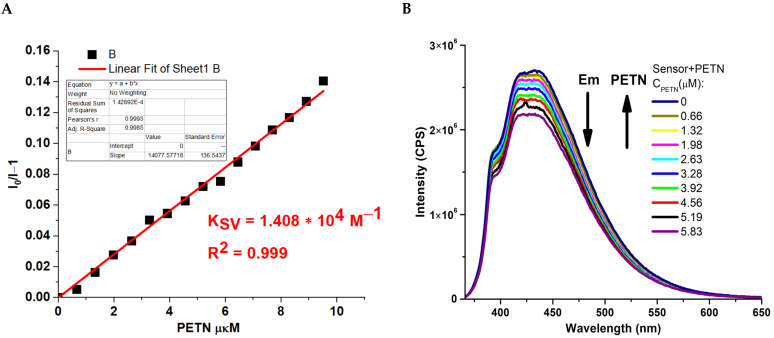
Stern–Volmer plot (**A**) and overlayed graph (**B**) of the chemosensor **3g** fluorescence quenching by PETN. PETN = Pentaerythritol tetranitrate.

The materials and devices for the remote detection of explosives, including fluorescence-based ones, are of high demand from the perspective of a high risk of terrorist attack worldwide [40,41,42]. Additionally, pyrene derivatives, including azole-appended ones, are known to exhibit a well-pronounced fluorescence “turn-off” response toward common nitroatomatic analytes [43,44,45,46,47,48], as well as some nitroaliphatic explosive components [49,50]. In addition, fluorescence- and aggregation-induced emission (AIE)-based small/single-molecule fluorophores, sensors, and probes [51,52,53,54] are of high demand for biovisualization applications [55,56,57,58,59,60,61,62]. Therefore, as a final step, we studied the ability of the present POPOP *aza*-analogs **3** to detect nitroanalytes. In acetonitrile solutions (10^−5^ M), except for pyrene derivatives **3c**,**g**, most of the fluorophores showed no response to both nitro-aromatic and nitro-aliphatic explosives. Compound **3g** exhibited a well-pronounced fluorescence “turn-off” response toward both traditional nitro-aromatic explosive components, such as TNT (K_sv_ = 12,036 M^−1^) and DNT (K_sv_ = 8427 M^−1^), and nitro-aliphatic explosive components, such as pentaerythritol tetranitrate (PETN, K_sv_ =14078 M^−1^, Figure 3), with a limit of detection (LOD) of 182 ppb for TNT and 183 ppb for PETN (Figure S1, Supplementary material).

The above-mentioned results are comparable to the most recent state-of-the-art studies [63,64,65,66]. The response of **3g** toward the above-mentioned nitro-explosives can be explained using a simple static or pseudo-static/false model when a nonradiative molecular complex “sensor—explosive” is formed in a ground state. In the case of compound **3c**, a nonlinear behavior is observed in the Stern–Volmer plot (see ESI), which can be explained by using a mixed static and dynamic Stern–Volmer quenching model.

Next, DFT studies [67,68,69,70,71] were carried out to explain the efficient detection of PETN by the compound **3g**. Thus, one can assume that the high sensitivity of **3g** to PETN lies in the possibility of an efficient photon-induced electron transfer (PET) from the LUMO of **3g** to the LUMO of PETN, which leads to the non-radiative decay of the exited state of the sensor. This process becomes possible if the LUMO of the sensor is much higher in energy. For the evaluation of the PETN quenching mechanism, quantum chemical calculations were carried out based on the B3LYP/def2-TZVP//PM6 level of theory with the help of the Gaussian-09 [72].

**Figure 4:**
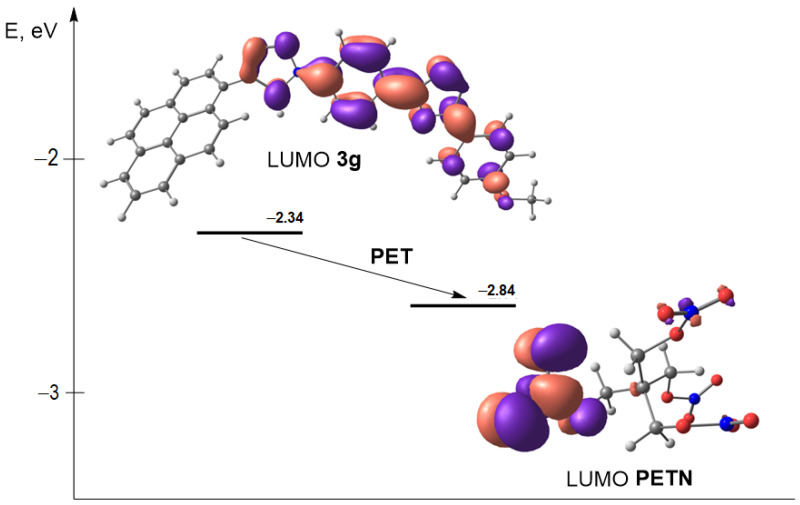
Energy diagram of the PET quenching process. PET = photon-induced electron transfer; PETN = Pentaerythritol tetranitrate.

In the case of the chemosensor/fluorophore **3g**, one can suggest a PET-emission quenching mechanism in the presence of PETN (as a quencher) [73,74,75]. Additionally, this suggestion is strongly supported by the fact that the LUMO energy of the sensor **3g** (−2.34 eV) is higher compared to the LUMO energy of the PETN quencher (−2.84 eV). Additionally, the calculated energy difference of 0.5 eV is a driving force of the quenching process (Table 2, Figure 4).

## 3. Materials and Methods

### 3.1. Synthesis

#### 3.1.1. 2-(4-Azidophenyl)-5-phenyl-1,3,4-oxadiazoles **2a**–**b**

General procedure. Corresponding 4-(5-aryl-1,3,4-oxadiazol-2-yl)aniline (1 equiv.) and para-toluenesulphonic acid monohydrate (1.05 equiv.) were dissolved in acetic acid at room temperature. Isopropyl nitrite (1.50 equiv.) added in one portion. After half an hour of stirring the sodium azide (1.50 equiv.) water solution added. After gas evolution ceased (diazonium probe negative), resulting suspension was filtered off and rinsed by water. Cake dried on air.

**2a**: Yield 240 mg, 92%. ^1^H NMR in DMSO-d_6_, δ, ppm: 7.37 (m, 2H); 7.65 (m, 3H), 8.15 (m, 4H). ^13^C NMR in DMSO-d_6_, ppm: 119.7 (1C), 120 (1C), 123.1 (1C), 126.4 (1C), 128.2 (1C), 129.1 (1C), 132 (1C), 143 (1C), 163.3 (1C), 164 (1C). EI-MS, *m*/*z* (I, %): 263 (11).

**2b**: Yield 563 mg, 100%. ^1^H NMR in DMSO-d_6_, δ, ppm: 3.87 (3H, s, CH_3_O-C_6_H_4_), 7.17 (m, 2H, CH_3_O-C_6_H_4_), 7.36 (m, 2H, CH_3_O-C_6_H_4_), 8.07 (m, 2H, C_6_H_4_), 8.13 (m, 2H, C_6_H_4_). ^13^C NMR in DMSO-d_6_, ppm: 115 (1C), 116 (1C), 116.3 (1C), 121 (1C), 121.2 (1C), 129 (1C),129.2 (1C), 143.4 (1C), 163 (1C), 164 (1C), 164.4 (1C). EI-MS, *m*/*z* (I, %): 293 (6).

#### 3.1.2. 2-Phenyl-5-(4-(4-arenyl-1H-1,2,3-triazol-1-yl)phenyl)-1,3,4-oxadiazoles **3a**–**g**

**Procedure A.** In 50 mL ace flask were successively dissolved in water (3 mL) sodium hydroxide (0.40 equiv.), ascorbic acid (0.50 equiv.) and copper (II) sulfate pentahydrate (0.20 equiv.). To the resulting milky suspension added a solution of corresponding etynyl derivative (1 equiv.) and an azido derivative (1.05 equiv.) in THF (6 mL). The flask was argon flushed and stirred under argon at 70 °C for 16 h. Reaction mass was diluted by aqueous 10% NH_4_OH (10 mL), the precipitated suspension filtered. Cake washed with water and air dried.

**Procedure B.** In 50 mL ace flask were successively added corresponding 2-(4-azidophenyl)-5-aryl-1,3,4-oxadiazole (1 equiv.), copper (I) bromide (0.20 equiv.) in the presence of triethylamine (2 equiv.), 1-ethynylpyrene (1.05 equiv.) were added to 5 mL of DMF. RM heated for 10 h at 100 °C in an argon atmosphere. After the reaction was completed (TLC monitoring), the reaction mass was diluted by aqueous 10% NH_4_OH (10 mL). The resulting suspension was filtered. Cake dried on air. Product was purified by flash chromatography if needed.

**3a** Procedure A. Yield 96%. ^1^H NMR in DMSO-d_6_, δ, ppm: 7.39 (m, 1H, Ph), 7.50 (m, 2H, Ph), 7.65 (m, 3H, Ph), 7.97 (m, 2H, Ph), 8.17 (m, 2H, Ph), 8.25 (d, 2H, J^3^ = 8.53 Hz, Ph), 8.38 (d, 2H, J^3^ = 8.53 Hz, Ph). ^13^C NMR in DMSO-d_6_, ppm: 30.3 (1C), 119.3 (1C), 120.3 (1C), 123.0 (1C), 125.2 (1C), 126.5 (1C), 128.1 (1C), 128.2 (1C), 129 (1C), 129.1 (1C), 130 (1C), 132 (1C), 139 (1C), 147.4 (1C), 163.1 (1C), 164.1 (1C). EI-MS, *m*/*z* (I, %): 365 (1).

**3b** Procedure A. The product was purified by flash chromatography (chloroform-silica gel). Yield 200 mg, 73%. ^1^H NMR in DMSO-d_6_, ppm: 7.58–7.72 (5,m, C_6_H_5_), 7.58–7.72 (m, 1H, C_10_H_7_), 7.91 (m, 1H, C_10_H_7_), 8.05 (m, 2H, C_10_H_7_), 8.19 (m, 2H, C_10_H_7_), 8.30–8.51 (m, 4H, C_6_H_4_), 8.58 (m, 1H, C_10_H_7_), 9.45 (s, 1H, C_2_N_3_H). ^13^C NMR in DMSO-d_6_, ppm: 60.1 (1C), 120.5 (1C), 122.1 (1C), 123.1 (1C), 125.2 (1C), 125.3 (1C), 126.1 (1C), 126.6 (1C), 126.6 (1C), 127.1 (1C), 128.2 (1C), 128.3 (1C), 128.8 (1C), 129.2(1C), 128.8 (1C), 131.9 (1C), 133.3 (1C), 138.0 (1C),138.1 (1C), 146.7 (1C), 163.1 (1C), 164.1 (1C). EI-MS, *m*/*z* (I, %): 415 (1).

**3c** Procedure B. Yield 158 mg, 85%. ^1^H NMR in DMSO-d_6_, ppm: 7.65(m, 3H, Ph), 8.06–8.50 (m, 14H, Ph + Pyr + C_2_N_3_H_4_), 8.96 (d, 1H, Pyr), 9.58 (s, 1H, C_2_N_3_H). ^13^C NMR in DMSO-d_6_, ppm: 121.2 (1C), 122.9 (1C), 123.4 (1C), 123.5 (1C), 124.0 (1C), 124.4 (1C), 124.7 (1C), 125.0 (1C),125.3 (1C), 125.5 (1C), 125.8 (1C), 126.7 (1C), 126.8 (1C), 127.0 (1C), 127.4 (1C), 127.5 (1C), 127.9 (1C), 128.1 (1C),128.4 (1C), 128.6 (1C), 129.6 (1C), 130.5 (1C), 131.1 (1C), 132.3 (1C), 139.0 (1C), 147.6 (1C), 163.5 (1C), 164.4 (1C). EI-MS, *m*/*z* (I, %): 489 (3).

**3d** Procedure B. Yield 138 mg, 70%. ^1^H NMR in DMSO-d_6_, ppm: 7.57–7.87 (m, 7H, Ph+ C_18_H_11_), 8.10–8.46 (m, 7H, Ph + C_6_H_4_),8.88 (m, 5H, C_18_H_11_), 9.31 (d, 1H, C_2_N_3_H), 972 (d, 1H, C_2_N_3_H). ^13^C NMR in DMSO-d_6_, ppm: 120.1 (1C), 120.2 (1C), 120.5 (1C), 123.3 (1C), 123.5 (1C), 124 (1C), 124.5 (1C), 125 (1C), 126.6 (1C), 128 (1C), 128.1 (1C), 128.4 (1C), 129 (1C), 129.1 (1C), 129.2 (1C), 129.3 (1C), 129.5 (1C), 130 (1C), 130.2 (1C), 131.1 (1C), 132.1 (1C), 139 (1C), 148 (1C), 163.3 (1C), 164.3 (1C). EI-MS, *m*/*z* (I, %): 515 (1).

**3e** Procedure B. Yield 133 mg, 99%. ^1^H NMR in DMSO-d_6_, ppm: 3.91 (s, 3H, CH_3_O-C_6_H_4_), 7.13 (m, 2H, CH_3_O-C_6_H_4_), 7.60 (m, 3H, C_10_H_7_), 7.88 (m, 1H, C_10_H_7_), 7.98 (2H, m, C_10_H_7_), 8.09 (2H, m, CH_3_O-C_6_H_4_), 8.35 (m, 4H, C_6_H_4_), 8.59 (m, 1H, C_10_H_7_), 9.36 (s, 1H, C_2_N_3_H). ^13^C NMR in DMSO-d_6_, ppm: 55.3 (1C),115 (1C), 115.35 (1C), 120.5 (1C),122.0 (1C), 123.1 (1C), 125.2 (1C), 125.3 (1C),126.0 (1C), 127 (1C), 127.0 (1C), 128.0 (1C), 128.2 (1C), 128.4 (1C), 129 (1C), 130.0 (1C), 134 (1C), 138.4 (1C), 147 (1C), 162.0 (1C), 163 (1C), 164.0 (1C). EI-MS, *m*/*z* (I, %): 395 (1).

**3f** Procedure B. Yield 122 mg, 80%. ^1^H NMR in DMSO-d_6_, ppm: 3.91 (s, 3H, CH_3_O-C_6_H_4_), 7.13 (m, 2H, CH_3_O-C_6_H_4_), 7.60 (m, 3H, C_10_H_7_), 7.88 (m, 1H, C_10_H_7_), 7.98 (m, 2H, C_10_H_7_), 8.09 (m, 2H, CH_3_O-C_6_H_4_), 8.35 (m, 4H, C_6_H_4_), 8.59 (m, 1H, C_10_H_7_), 9.36 (s, 1H, C_2_N_3_H). ^13^C NMR in DMSO-d_6_, ppm: 55.3 (1C),115 (1C), 115.35 (1C),120.5 (1C),122.0 (1C), 123.1 (1C), 125.2 (1C), 125.3 (1C),126.0 (1C), 127 (1C), 127.0 (1C), 128.0 (1C), 128.2 (1C), 128.4 (1C), 129 (1C), 130.0 (1C), 134 (1C), 138.4 (1C), 147 (1C), 162.0 (1C), 163 (1C), 164.0 (1C). EI-MS, *m*/*z* (I, %): [M-N_2_]^+^ = 417 (18).

**3g** Procedure B. Yield 143 mg, 50%. ^1^H NMR in DMSO-d_6_, ppm: 3.91 (s, 3H, CH_3_O-C_6_H_4_), 7.14 (m, 2H, CH_3_O-C_6_H_4_), 8.07-8.14 (m, 3H, Ph + Pyr + C_2_N_3_H_4_), 8.16-8.32 (m, 5H, Ph + Pyr + C_2_N_3_H_4_), 8.34-8.46 (m, 4H, C_6_H_4_), 8.34-8.46 (m, 2H, CH_3_O-C_6_H_4_),8.96 (d, 1H, Pyr), 9.55 (s, 1H, C_2_N_3_H). ^13^C NMR in DMSO-d_6_, ppm: 55.5 (1C), 95.3 (1C), 115 (1C), 115.3 (1C), 115.5 (1C), 121 (1C), 123 (1C), 123.4 (1C),124 (1C), 124.2 (1C), 124.5 (1C), 125 (1C), 125.1 (1C), 125.3 (1C), 126 (1C), 126.5 (1C), 127.3 (1C), 128 (1C), 128.3 (1C), 129(1C), 130.3 (1C), 131 (1C),138 (1C),139 (1C),147.4 (1C), 162.2 (1C), 163 (1C), 164.2(1C). EI-MS, *m*/*z* (I, %): [M-N_2_]^+^ = 491 (12).

For the NMR spectra of the synthesized fluorophores (**3a**–**g**) please see the Supplementary material (Figures S2–S10).

### 3.2. Photophysical Investigations

#### Materials and Equipment

Acetonitrile and methylene chloride were used to prepare a solution of POPOP azaanalogues in order to study the photophysical properties, purity levels “for HPLC, UV, IR, GPC”. Absorption spectra were recorded on a Shimadzu UV-1800 spectrophotometer (Kyoto, Japan). The emission and excitation spectra were recorded on a Horiba-FluoroMax-4 spectrofluorometer (Irvine, CA 92618, USA). Graphical processing of the absorption, emission and excitation spectra was performed using OriginPro 2015 (64-bit) b9.2.196 software. The absolute quantum yields of the photoluminescence of the compounds were obtained using the integrating sphere of the Horiba-Fluoromax-4 instrument (Irvine, CA 92618, USA). Graphical processing of absorption, emission and excitation spectra was carried out using OriginPro 2015 (64-bit) b9.2.196 software; normalization of all electronic spectra was carried out in the Overlay mode automatically using the “Normalize columns” option using the same software. The absolute quantum yields of the photoluminescence of the compounds were obtained using the integrating sphere of the Horiba-Fluoromax-4 instrument (Irvine, CA 92618, USA). The fluorescence lifetime of the compounds was measured on a Horiba FluoroMax-4 instrument (USA) using the TCSPC (Time Correlated Single-Photon Counting) method.

**Figure 5:**
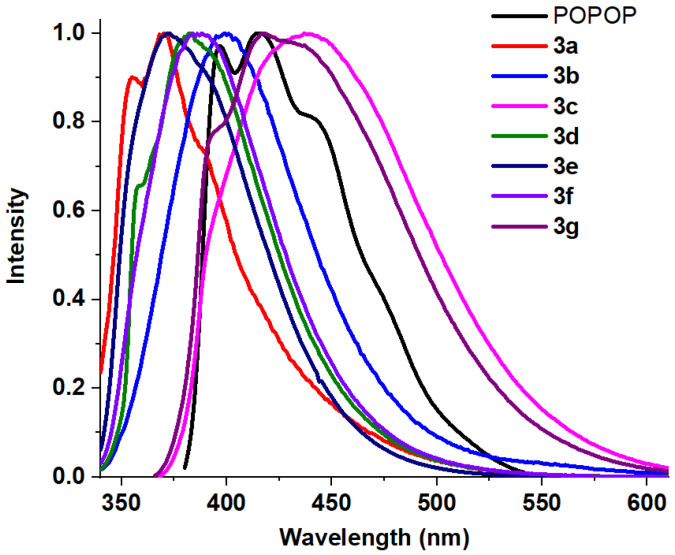
Normalized emission spectra of aza analogues POPOP. POPOP = 1,4-Bis(5-phenyl-2-oxazolyl)benzene.

We chose the maxima closest to 350 nm, because this wavelength is the most preferred excitation wavelength when recording the emission spectrum, according to the operating instructions for the Horiba FluoroMax-4 spectrofluorometer (Figure 5).

### 3.3. Experimental Methods

#### 3.3.1. Fluorometric Titration

Compound **3g** was studied as a chemosensor for “turn-off” fluorescence detection of explosives. The chemosensor fluorescence response to nitroanalytes was quantified using the Stern-Volmer static quenching model. The Stern-Volmer fluorescence quenching constants (K_sv_) were calculated according to the static quenching equation as the slope of the intensity graph ((I_0_/I)^−1^) depending on the concentration of the quencher ([Q]) (Equation (1)):

(1) $$\frac{I_{0}}{I}=1+K_{SV}\left[Q\right]$$

Electron-deficient neutral molecules were chosen as quenchers—one of the most common explosives and their decay products 2,4,6-trinitrotoluene (TNT) and 2,4-dinitrotoluene (DNT), tetranitropentaerythritol (PETN) (2 × 10^−4^ M). Concentration **3g** (10^−6^ M) (Figure 6, Figure 7, Figure 8, Figure 9, Figure 10, Figure 11, Figure 12, Figure 13 and Figure 14).

#### 3.3.2. Limit of Detection Calculation

The limit of detection (LOD) was calculated on the basis of the data of fluorometric titration experiments according to the method published previously [76]. A calibration curve was plotted between the fluorescence intensity and the quencher concentration to obtain a regression curve equation. The LOD was determined using the Equation (2):

(2) LOD=3σ÷k

where σ is the standard deviation of the fluorophore intensity in the absence of an analyte obtained via STEYX function in Excel and k is the slope of the calibration curve.

#### 3.3.3. Time-Resolved Fluorescence Measurement

**Figure 15:**
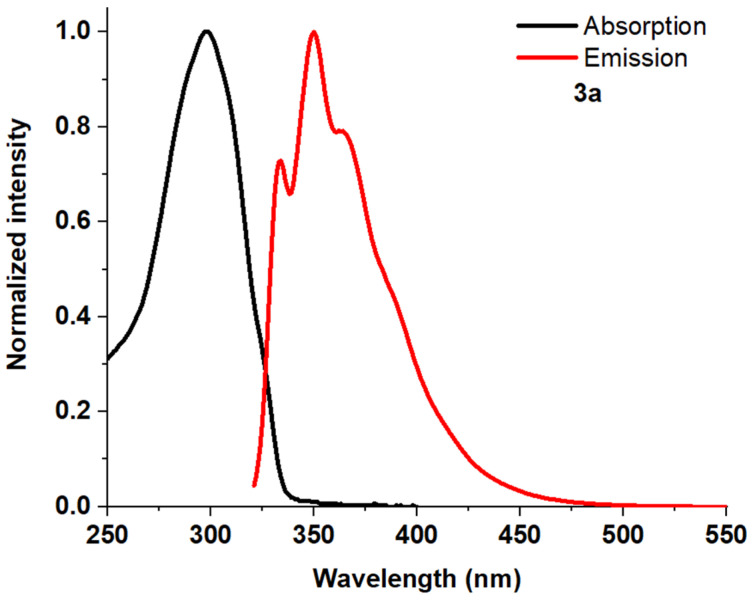
Normalized emission and absorption spectra **3a**.

**Figure 16:**
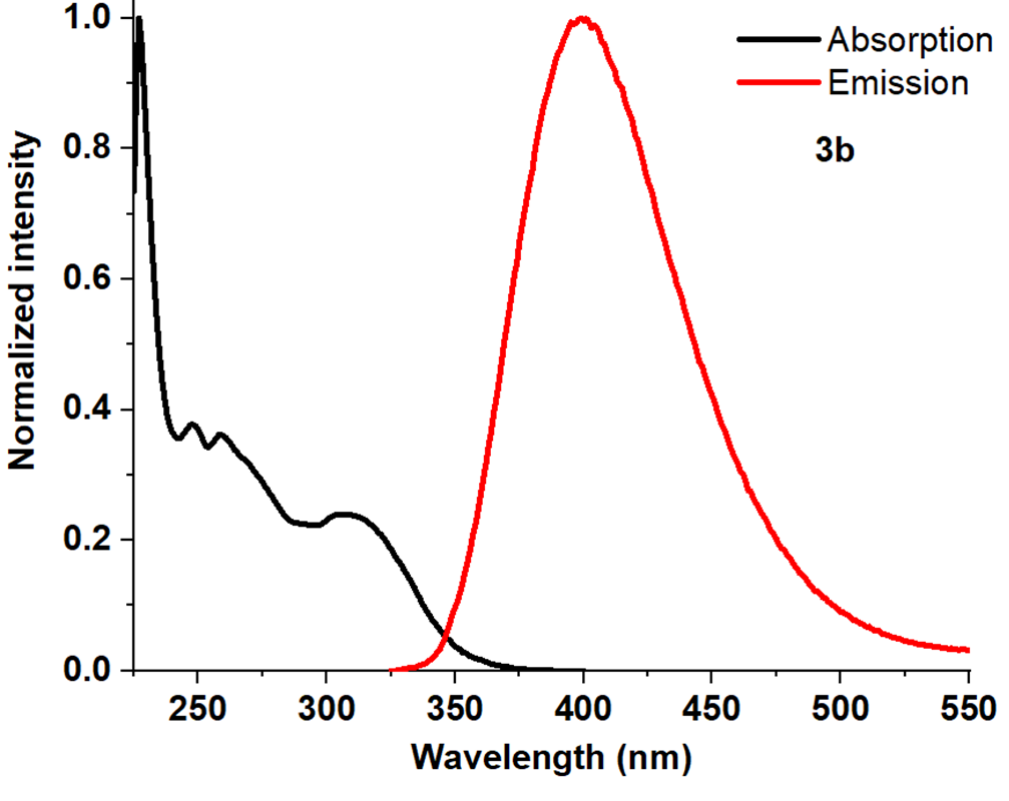
Normalized emission and absorption spectra **3b**.

**Figure 17:**
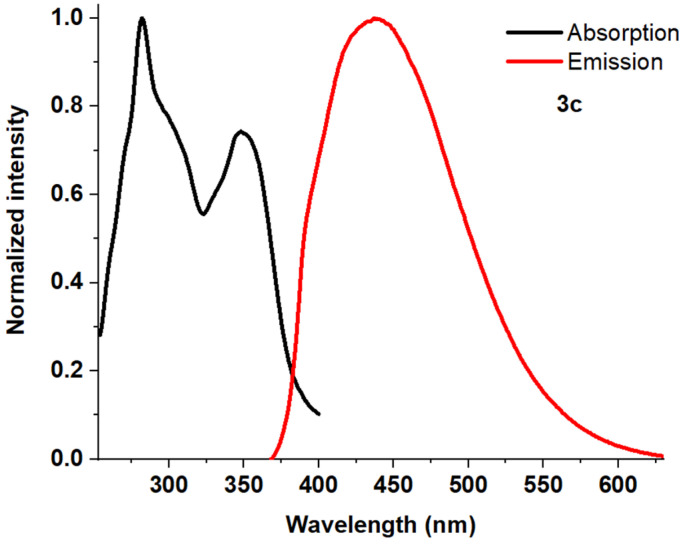
Normalized emission and absorption spectra **3c**.

**Figure 18:**
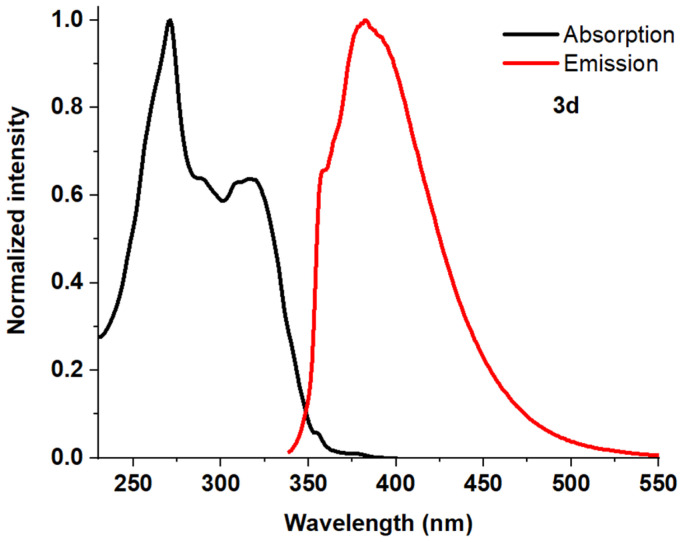
Normalized emission and absorption spectra **3d**.

**Figure 19:**
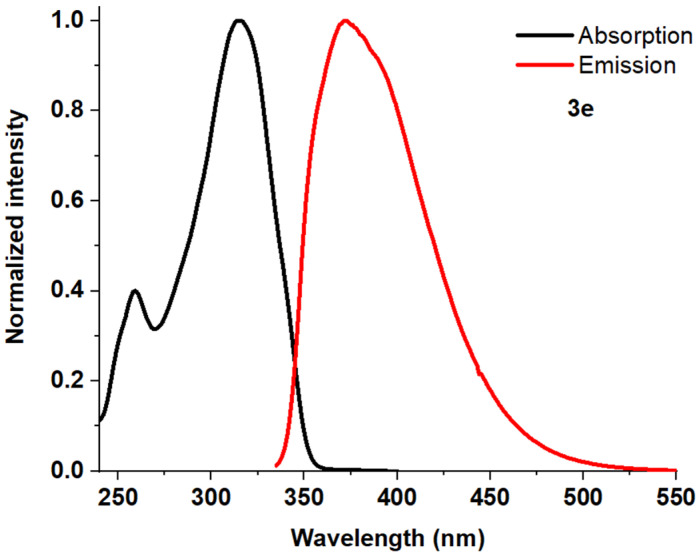
Normalized emission and absorption spectra **3e**.

**Figure 20:**
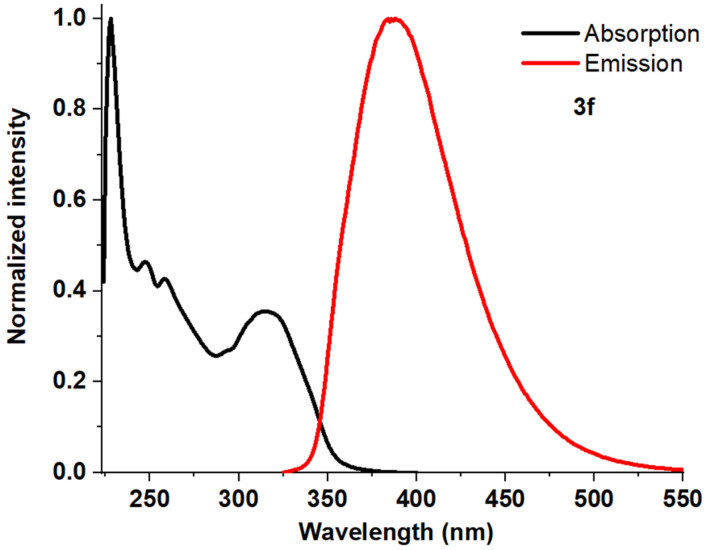
Normalized emission and absorption spectra **3f**.

**Figure 21:**
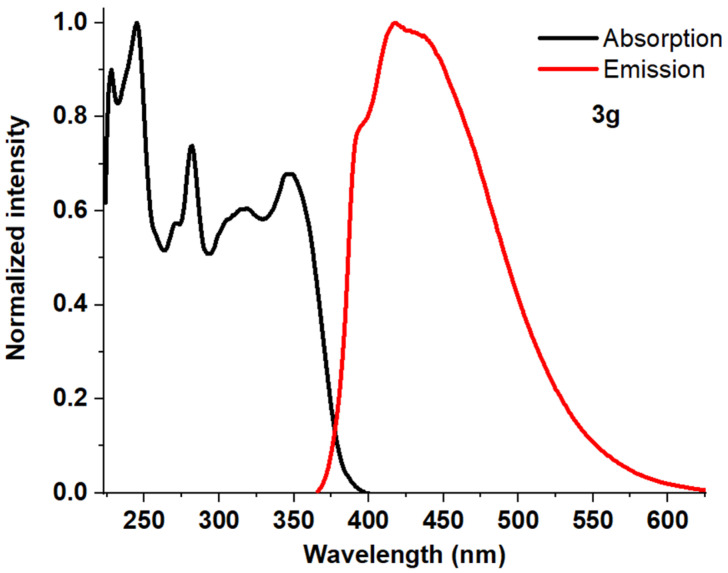
Normalized emission and absorption spectra **3g**.

We have also measured the time-resolved fluorescence of all the fluorophores **3**, which are summarized in Table 3 and Figure 15, Figure 16, Figure 17, Figure 18, Figure 19, Figure 20 and Figure 21.

#### 3.3.4. Excitation Spectra

**Figure 22:**
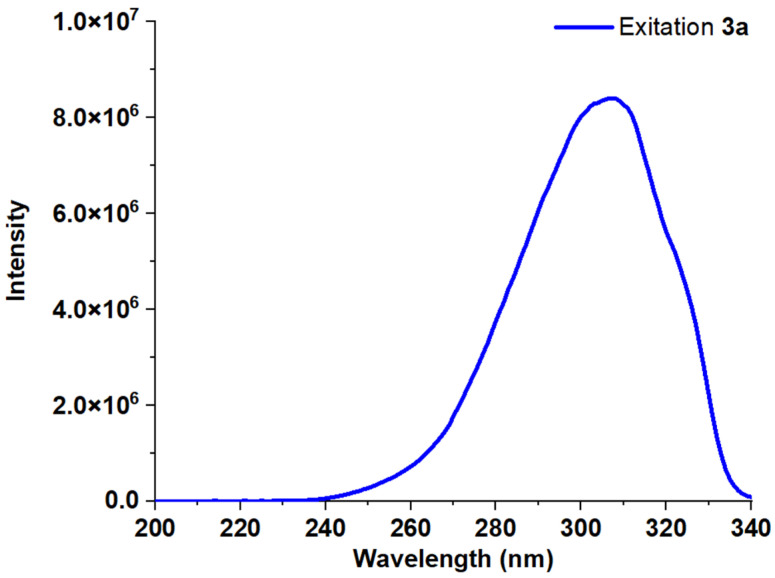
Excitation spectrum **3a**.

**Figure 23:**
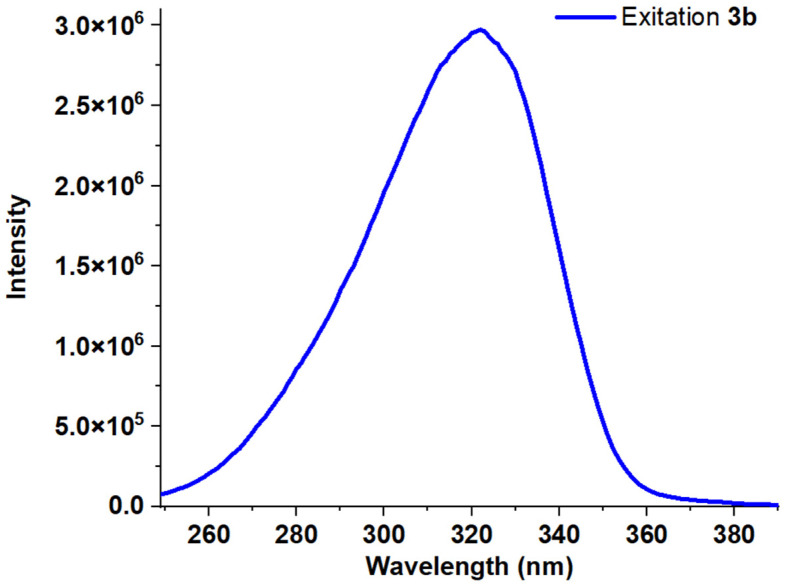
Excitation spectrum **3b**.

**Figure 24:**
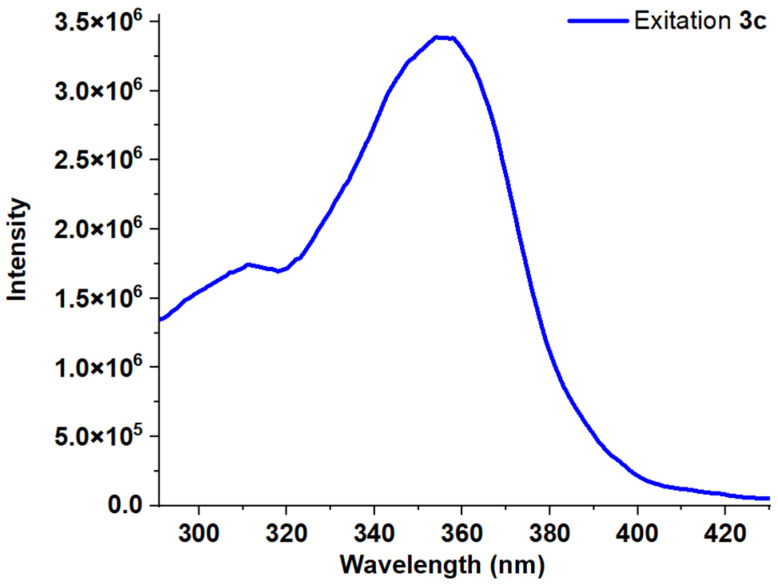
Excitation spectrum **3c**.

**Figure 25:**
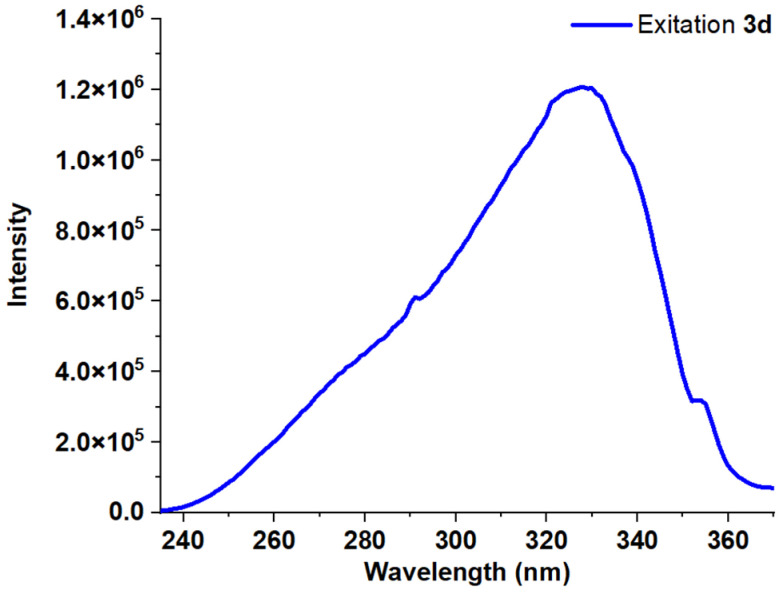
Excitation spectrum **3d**.

**Figure 26:**
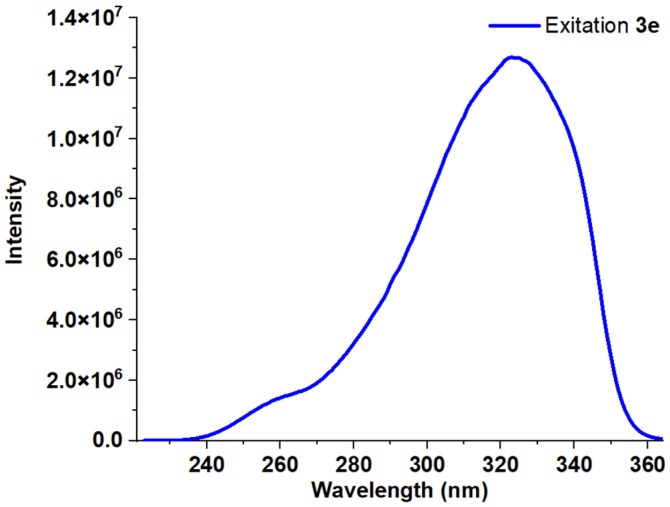
Excitation spectrum **3e**.

**Figure 27:**
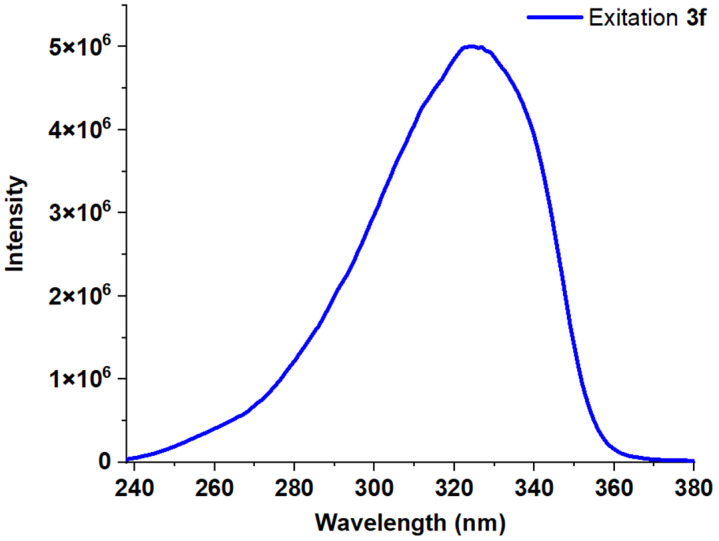
Excitation spectrum **3f**.

**Figure 28:**
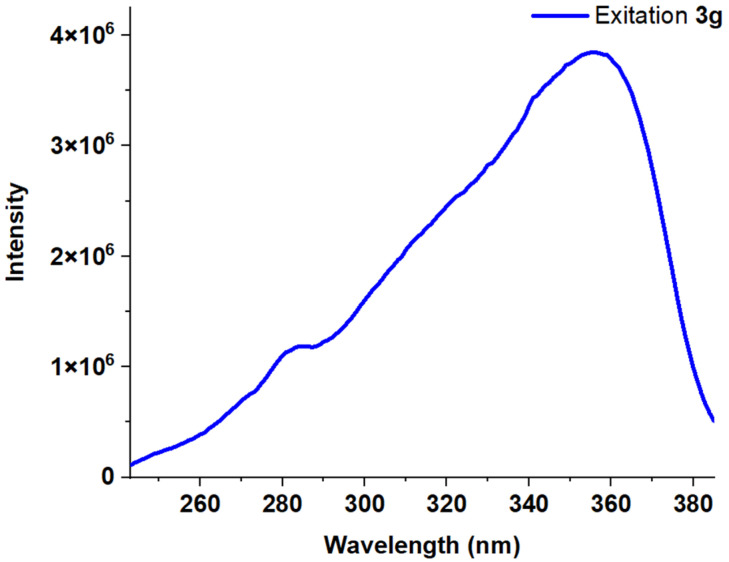
Excitation spectrum **3g**.

For all compounds, the excitation spectra were additionally measured in CH_2_Cl_2_ [10^−5^ M] (Figure 22, Figure 23, Figure 24, Figure 25, Figure 26, Figure 27 and Figure 28), at the emission wavelength. The resulting excitation spectra resemble the absorption spectra of the corresponding compounds. A significant decrease in the intensity of short-wavelength excitation bands is observed, which also correlates with the previously reported data [18].

## 4. Conclusions

In summary, PAH-based POPOP *aza*-analogues were successfully prepared by means of Cu(I)-catalyzed click reaction between 2-(4-azidophenyl)-5-Ar-1,3,4-oxadiazole and terminal ethynyl-substituted PAHs. Among all the obtained compounds, pyrenyl-substituted fluorophores, such as **3c**,**g**, exhibited the most promising photophysical properties, such as emission up to 441 nm and quantum yield up to 84%, which were closest to the ones reported for POPOP. In most cases, the introduction of an electron-donating methoxy group in the aromatic moiety of these *aza*-analogues of POPOP improved their photophysical properties. Among the obtained compounds, only pyrene-substituted fluorophore **3g** exhibited a well-pronounced fluorescence “turn-off” response toward several common nitroaromatic explosive components, such as DNT and TNT, with 0.8–1.2 × 10^4^ M^−1^ Stern–Volmer (quenching) constants and an LOD of 182 ppb for TNT. In addition, this compound exhibited an excellent response to the hard-to-detect nitro-aliphatic explosive, PETN, with a 1.4 × 10^4^ M^−1^ Stern–Volmer constant and an LOD of 183 ppb. Possible quenching via the PET mechanism for **3g** was suggested, and this was further supported by means of DFT quantum chemical calculations.

## Figures and Tables

**Figure 6:**
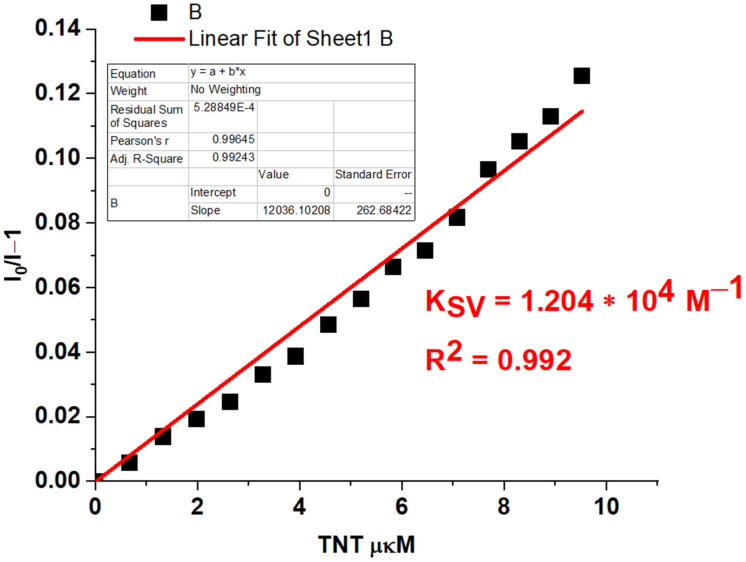
Fluorescence quenching of chemosensor **3g** by TNT. TNT = 2,4,6-Trinitrotoluene.

**Figure 7:**
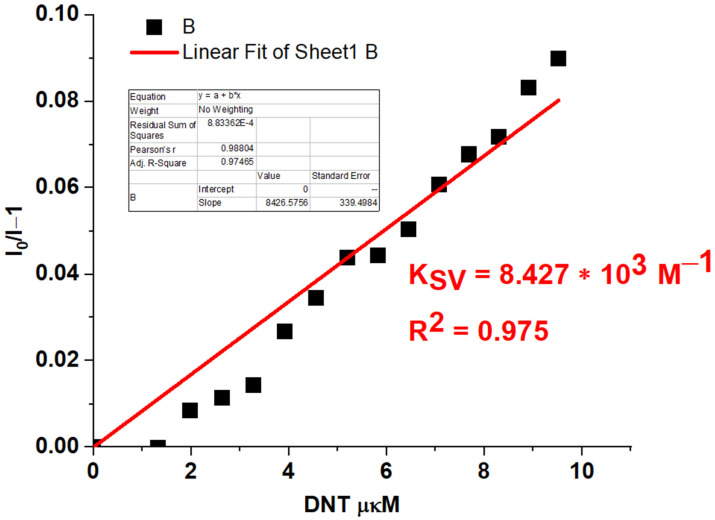
Fluorescence quenching of chemosensor **3g** by DNT. DNT = 2,4-Dinitrotoluene.

**Figure 8:**
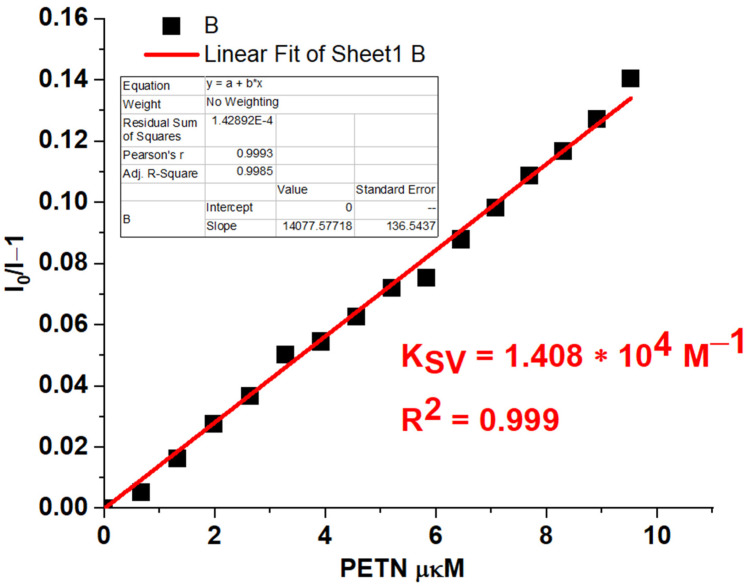
Fluorescence quenching of chemosensor **3g** by PETN. PETN = Pentaerythritol tetranitrate.

**Figure 9:**
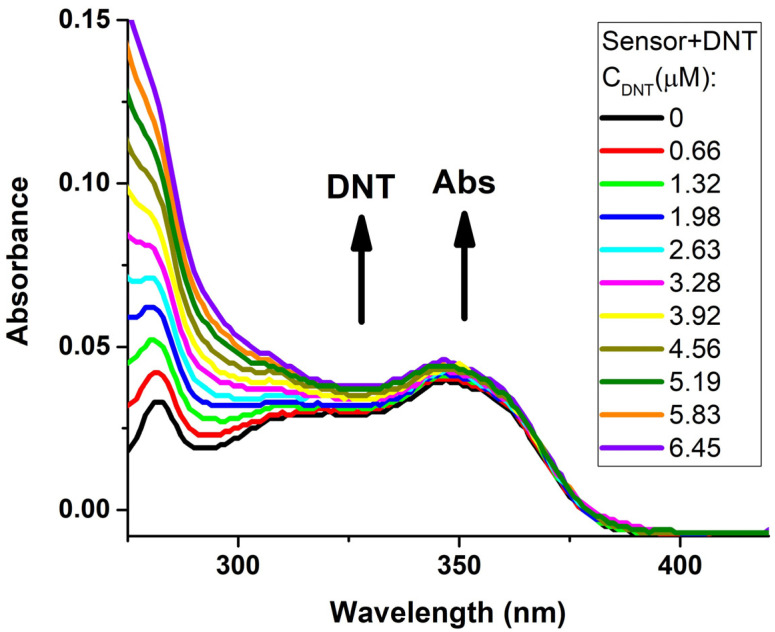
Overlayed graph of the chemosensor **3g** quenching by DNT (UV-Vis). DNT = 2,4-Dinitrotoluene.

**Figure 10:**
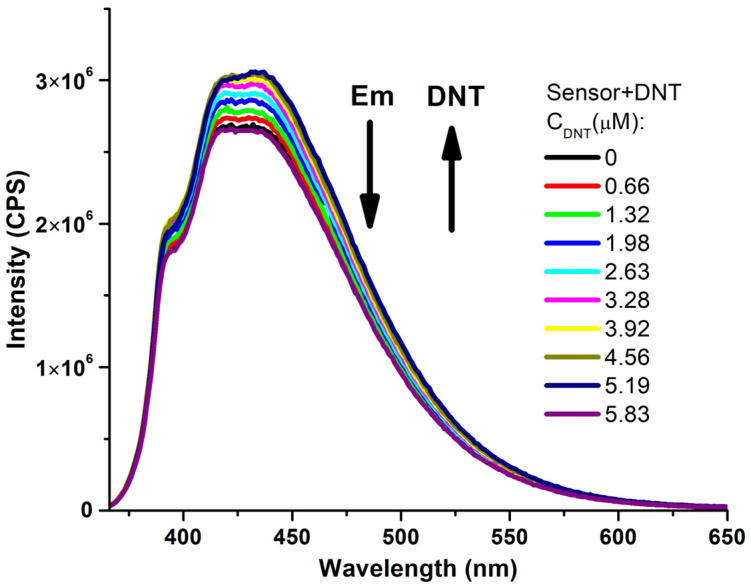
Overlayed graph of the chemosensor **3g** quenching by DNT (Emission). DNT = 2,4-Dinitrotoluene.

**Figure 11:**
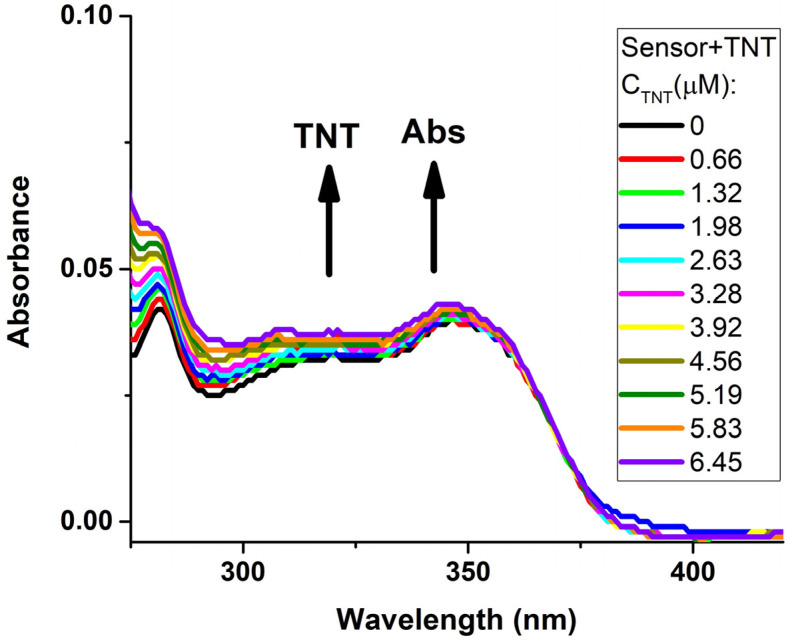
Overlayed graph of the chemosensor **3g** quenching by TNT (UV-Vis). TNT = 2,4,6-Trinitrotoluene.

**Figure 12:**
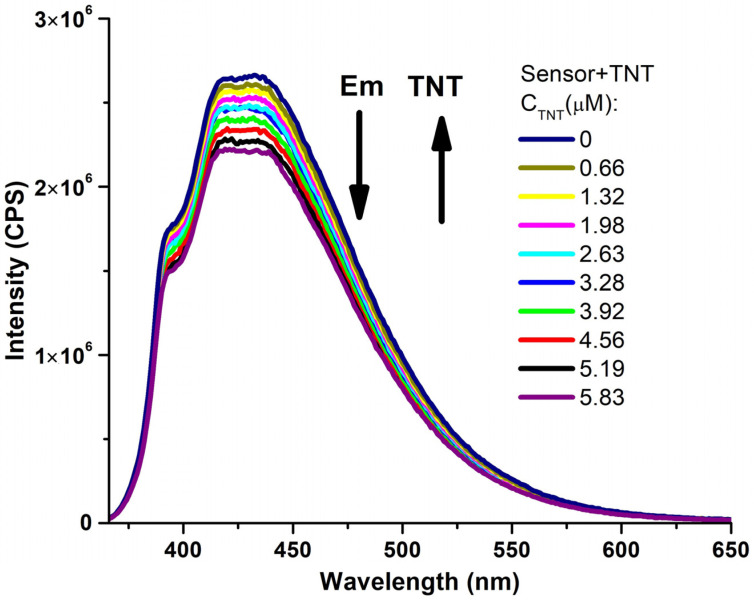
Overlayed graph of the chemosensor **3g** quenching by TNT (Emission). TNT = 2,4,6-Trinitrotoluene.

**Figure 13:**
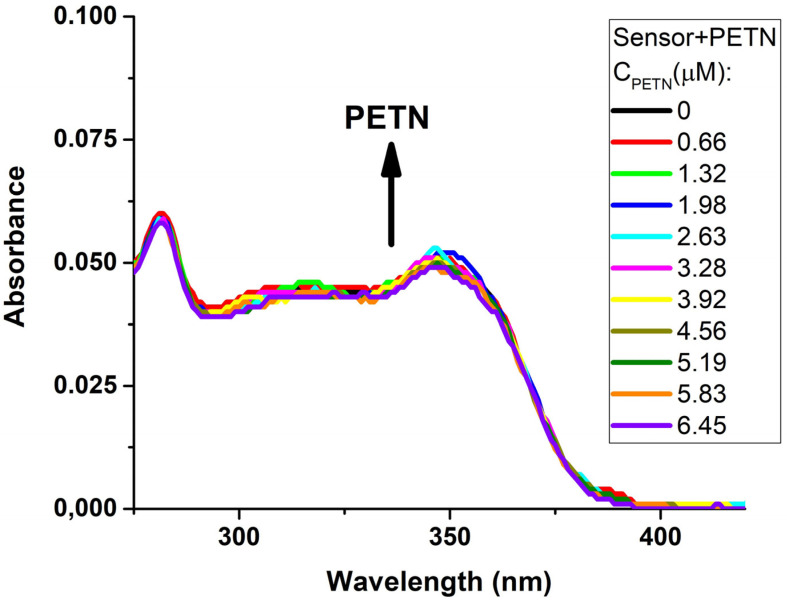
Overlayed graph of the chemosensor **3g** quenching by PETN (UV-Vis). PETN = Pentaerythritol tetranitrate.

**Figure 14:**
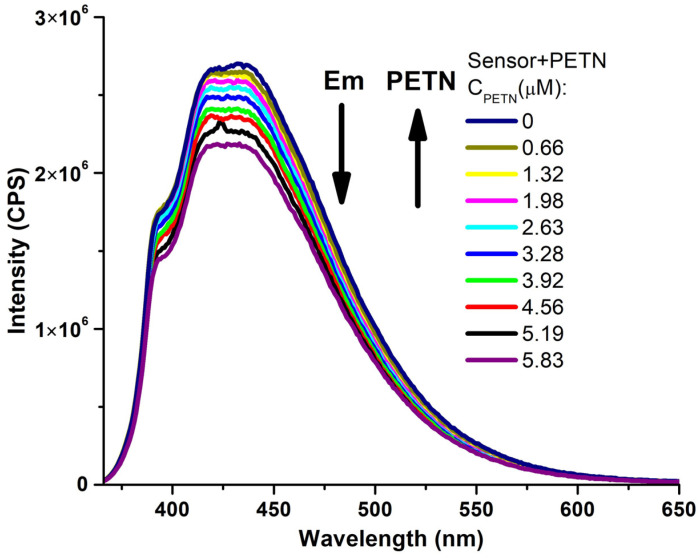
Overlayed graph of the chemosensor **3g** quenching by PETN (Emission). PETN = Pentaerythritol tetranitrate.

**Table 1:** Data showing the photophysical properties of fluorophores (**3**) and POPOP (10^−5^ M) in CH_2_Cl_2_ solvent.

Entry	Comp.	λ_abs max_ ^a^, nm (ε_M_, M^−1^ cm^−1^)	λ_em max_ ^b^, nm	Stokes Shift, nm	τ, ns ^c^	*Φ_f_*, (%) ^d^
1	**POPOP**	346 (99,000) 361 (111,400) 384 (68,000)	416 443	97		97.5 ^e^
2	**3a**	248 (43,300) 309 (61,800)	356 370	47		17
3	**3b**	248 (22,500) 259 (21,600) 307 (14,500)	398	150	0.66	48
4	**3c**	245 (17,800) 282 (35,600) 348 (26,300)	441	159	4.52	23
5	**3d**	271 (56,200) 317 (35,900)	382	111	1.22	30
6	**3e**	259 (31,500) 315 (75,200)	373	58	0.72	98.13 ^f^
7	**3f**	228 (93,400) 247 (43,400) 258 (39,900) 315 (33,200)	388	160	0.49	71
8	**3g**	228 (39,100) 245 (43,200) 282 (32,400) 319 (26,900) 346 (29,900)	393 (sh) 417	172	4.34	84

^a^ Absorption spectra were measured at r.t. in CH_2_Cl_2_ in the range from 200 to 450 nm; ^b^ emission spectra were measured at r.t. in CH_2_Cl_2_; ^c^ weighted average decay time τ_av_ = Σ (τi × αi) in CH_2_Cl_2_ (LED 310 nm); ^d^ absolute quantum yields were measured as reported earlier using the Integrating Sphere on a Horiba-Fluoromax-4 at r.t. in CH_2_Cl_2_; ^e^ calorimetric value in cyclohexane [3]; and ^f^ calorimetric value in THF.

**Table 2:** HOMO–LUMO energy levels for **3g** and PETN (in eV).

Structure	HOMO Energy	LUMO Energy
**3g**	−5.71	−2.34
PETN	−9.14	−2.84

**Table 3:** Fluorescence lifetime of probes **3a**–**g** (C = 2 × 10^−6^ M) in CH_2_Cl_2_.

Entry	Compound	τ_1_, ns ^a^	α_1_^b^	τ_2_, ns ^a^	α_2_^b^	τ_av_^c^, ns ^a^	χ^2 d^
1	**3a**	0.97	1.0000	-	-	0.97	1.08
2	**3b**	0.54	0.8942	1.64	0.1058	0.66	1.22
3	**3c**	1.55	0.1114	4.89	0.8886	4.52	1.15
4	**3d**	0.89	0.9092	4.56	0.0908	1.22	1.23
5	**3e**	0.72	1.0000	-	-	0.72	1.15
6	**3f**	0.42	0.9458	1.68	0.0542	0.49	1.18
7	**3g**	1.08	0.0672	4.58	0.9328	4.34	1.12

^a^ Decay time, ^b^ Fractional contribution, ^c^ Weighted average decay time τ_av_ = Σ (τ_i_ × α_i_), ^d^ Quality of fitting.

## Data Availability

Not applicable.

## Supplementary Materials

The following supporting information can be downloaded at https://www.mdpi.com/article/10.3390/ijms241210084/s1.
