# Supplementary material for: Polyaromatic Hydrocarbon (PAH)-Based Aza-POPOPs: Synthesis, Photophysical Studies, and Nitroanalyte Sensing Abilities
Source: Int J Mol Sci. 2023 Jun 13;24(12):10084. doi: 10.3390/ijms241210084 (PMC10298922; doi:10.3390/ijms241210084)
Supplement: Supplementary file 1 [file ijms-24-10084-s001.zip › ijms-2377658-supplementary.pdf]

Polyaromatic hydrocarbon (PAH) aza analogues of POPOP: synthesis and photophysical properties

Mohammed S. Mohammed,[1] Igor S. Kovalev,[1] Natalya V. Slovesnova,[1,2], Leila K. Sadieva,[1,3] Vadim A. Platonov,[1] Alexander S. Novikov,[4,5] Sougata Santra[1], Julia E. Morozova[6]*, Grigory V. Zyryanov,[1,3] Valery N. Charushin,[1,3] Brindaban C. Ranu[1,7]

[1] Ural Federal University, 19, Mira St., Yekaterinburg, 620002, Russian Federation

[2] Ural Medical University, 3, Repina St., Yekaterinburg, 620028, Russian Federation

[3] I. Ya. Postovsky Institute of Organic Synthesis of RAS (Ural Division), 22/20, S. Kovalevskoy/Akademicheskaya St., Yekaterinburg, 620137, Russian Federation

[4] Institute of Chemistry, Saint Petersburg State University, Universitetskaya Nab., 7/9, Saint Petersburg, 199034, Russian Federation

[5] Peoples' Friendship University of Russia (RUDN University), Miklukho-Maklaya St., 6, Moscow, 117198, Russian Federation

[6] Arbuzov Institute of Organic and Physical Chemistry, FRC Kazan Scientific Center of RAS, Arbuzov Str. 8, 420088 Kazan, Russian Federation

[7] School of Chemical Sciences, Indian Association for the Cultivation of Science, Jadavpur, Kolkata – 700 032, India

Contents

Chemical structures of the tested electron deficient compounds included in composition of explosives ..2

NMR spectra 3

Chemical structures of the tested electron deficient compounds included in composition of explosives

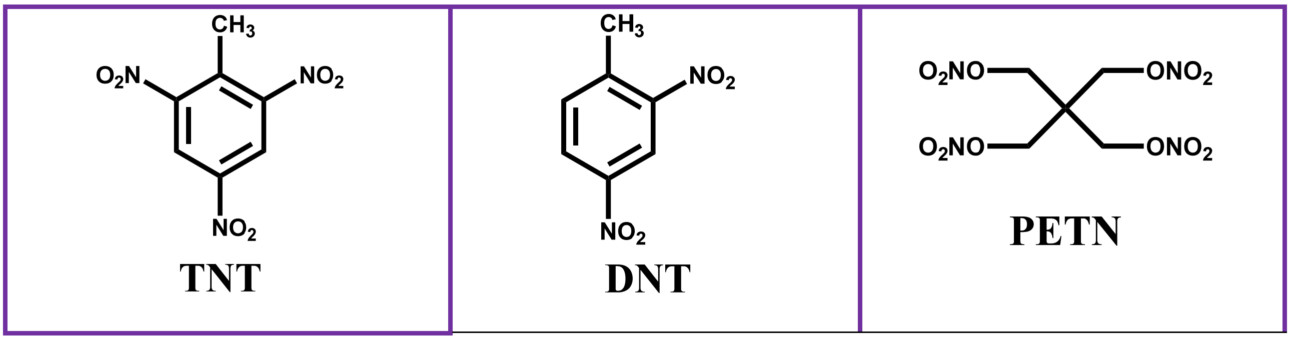

Figure S1. Chemical structures of 2,4,6-trinitrotoluene (TNT), 2,4-dinitrotoluene (DNT), pentaerythritol tetranitrate (PETN).

NMR spectra

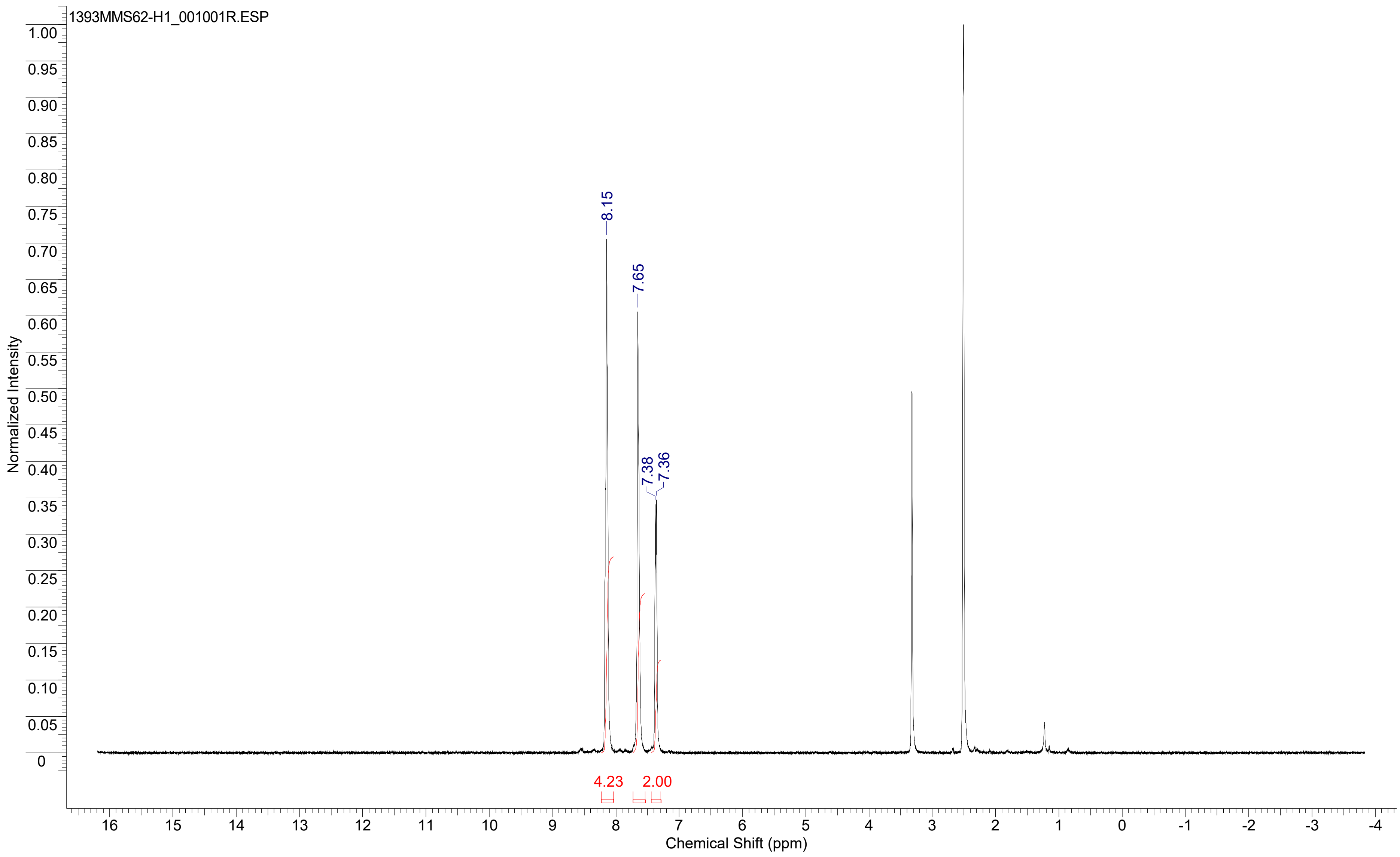

1393MMS62-H1_001001R.ESP
Normalized Intensity
8.15
7.65
7.38
7.36
4.23
2.00
Chemical Shift (ppm)

Figure S2. ^{1}H NMR spectrum of compound **2a**.

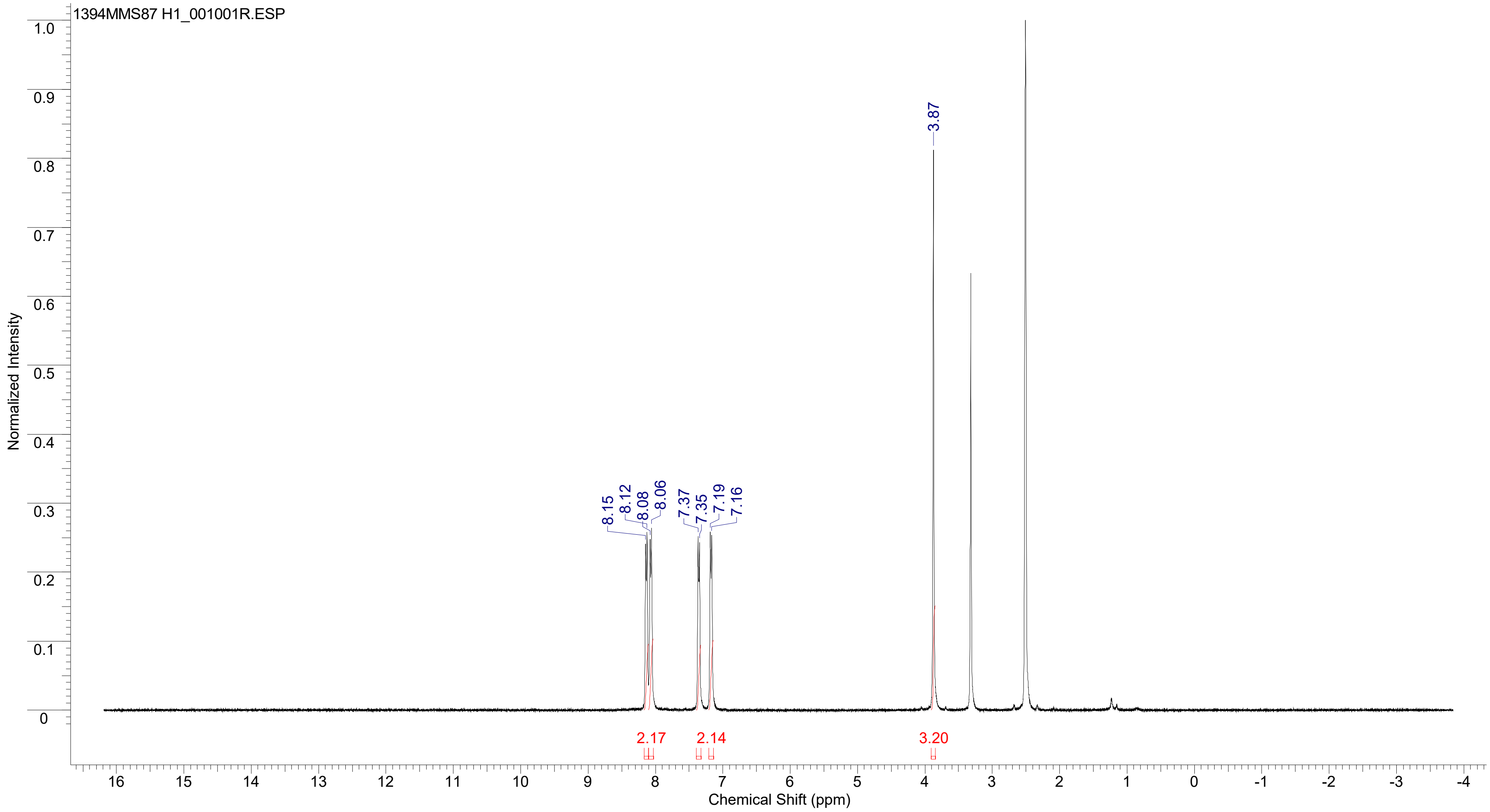

Figure S3. ^{1}H NMR spectrum of compound **2b**.

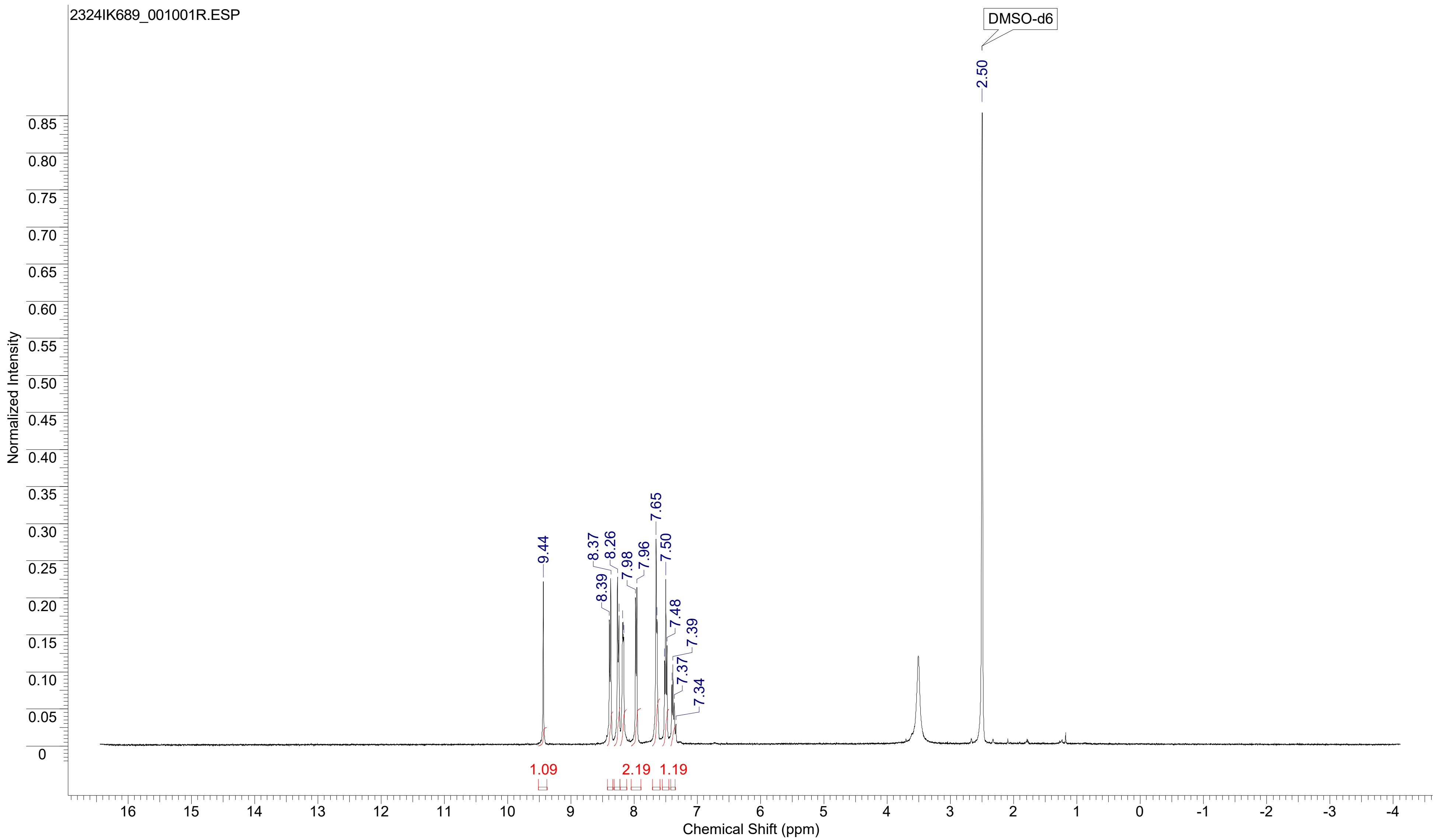

Figure S4. ^{1}H NMR spectrum of compound **3a**

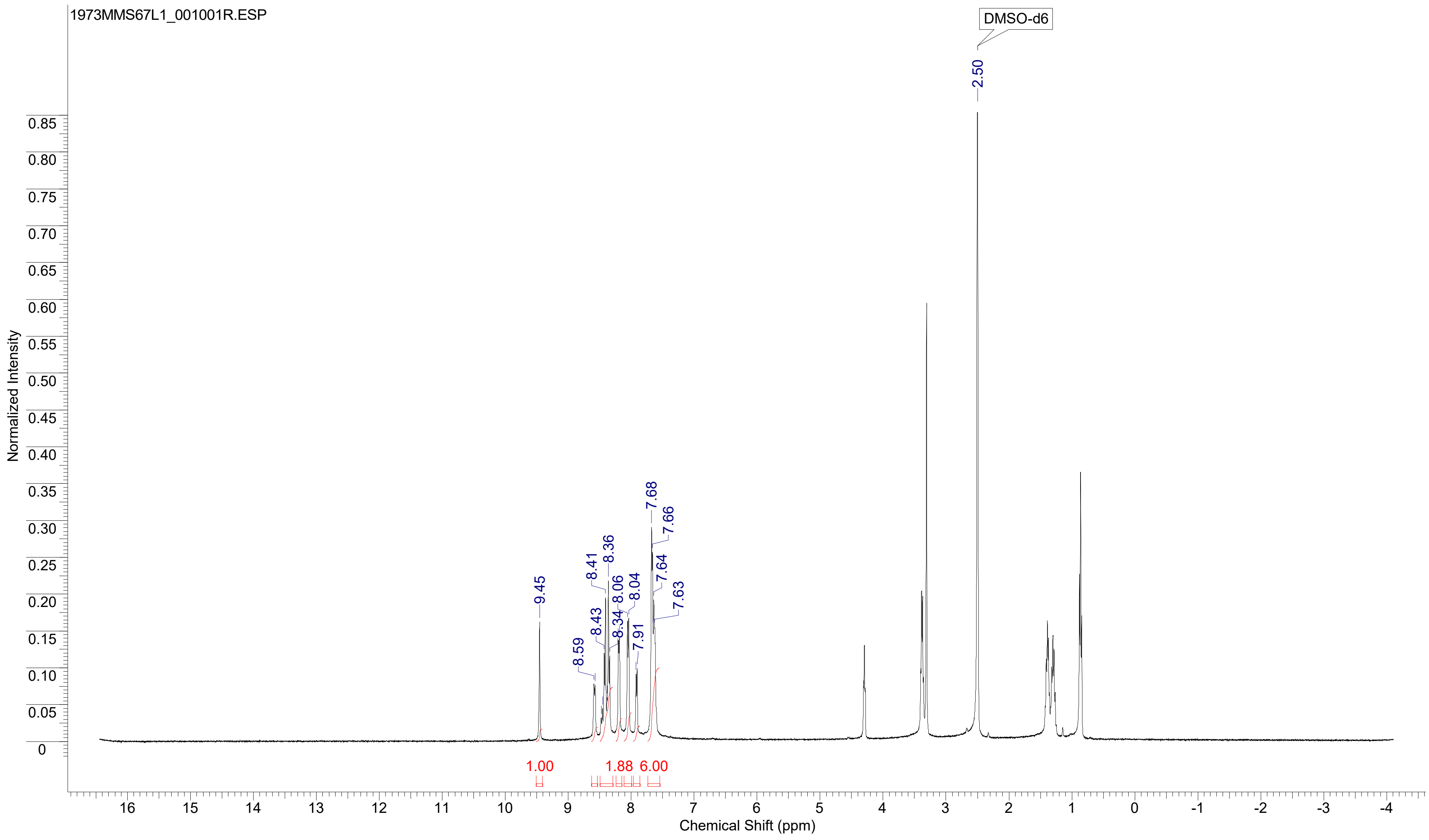

Figure S5. ^{1}H NMR spectrum of compound **3b**.

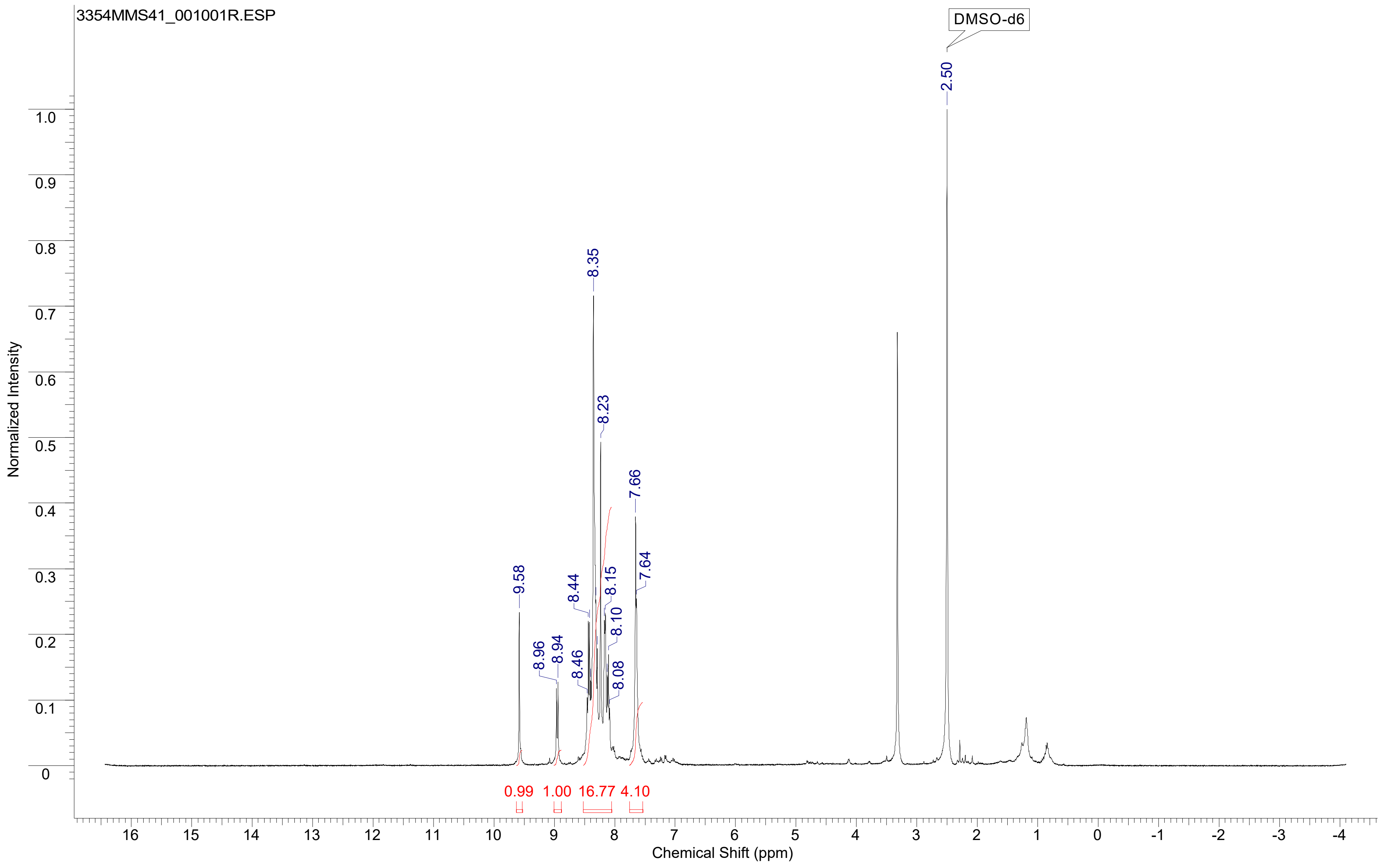

Figure S6. ^{1}H NMR spectrum of compound **3c**.

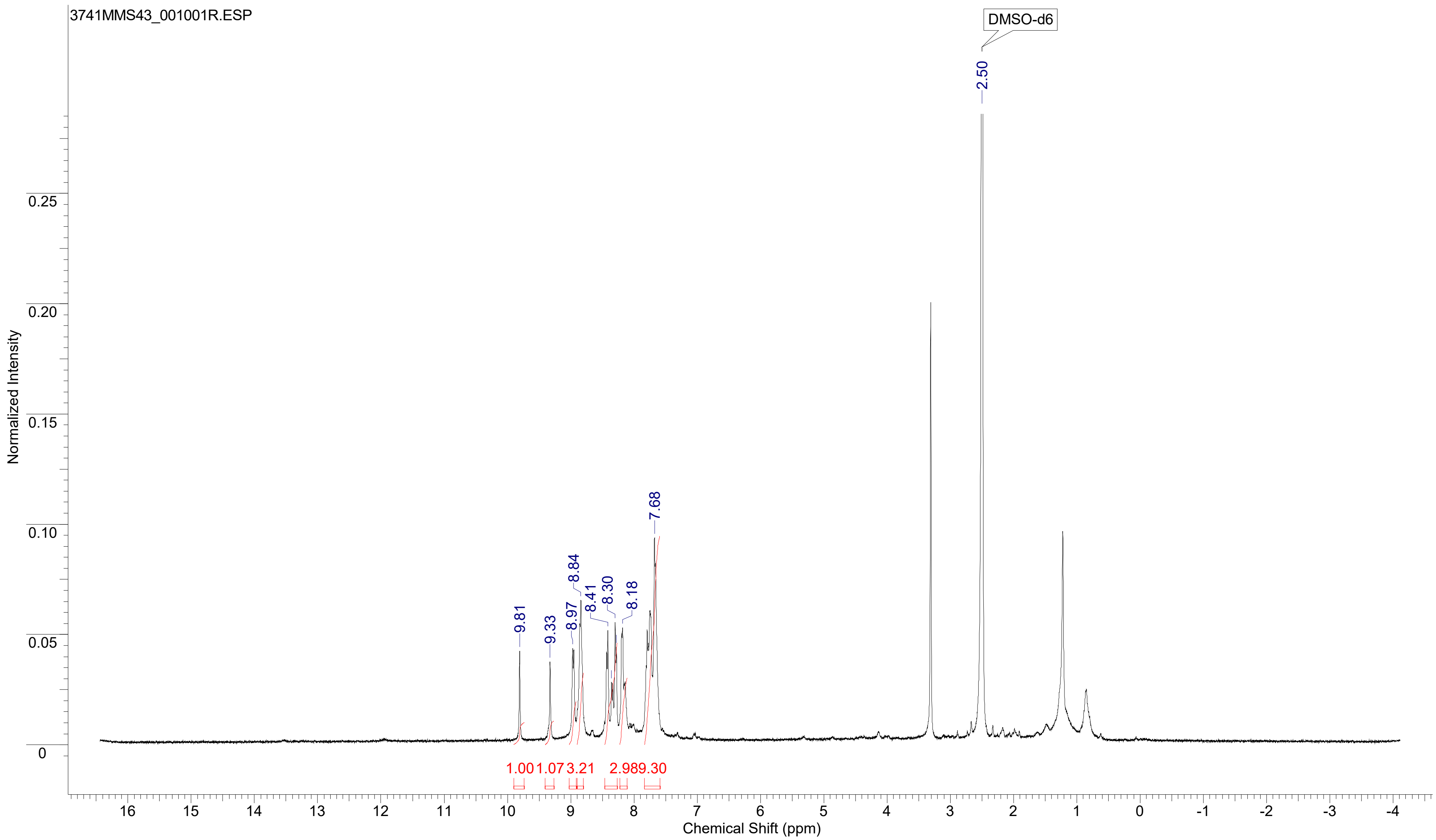

Figure S7. ^{1}H NMR spectrum of compound **3d**.

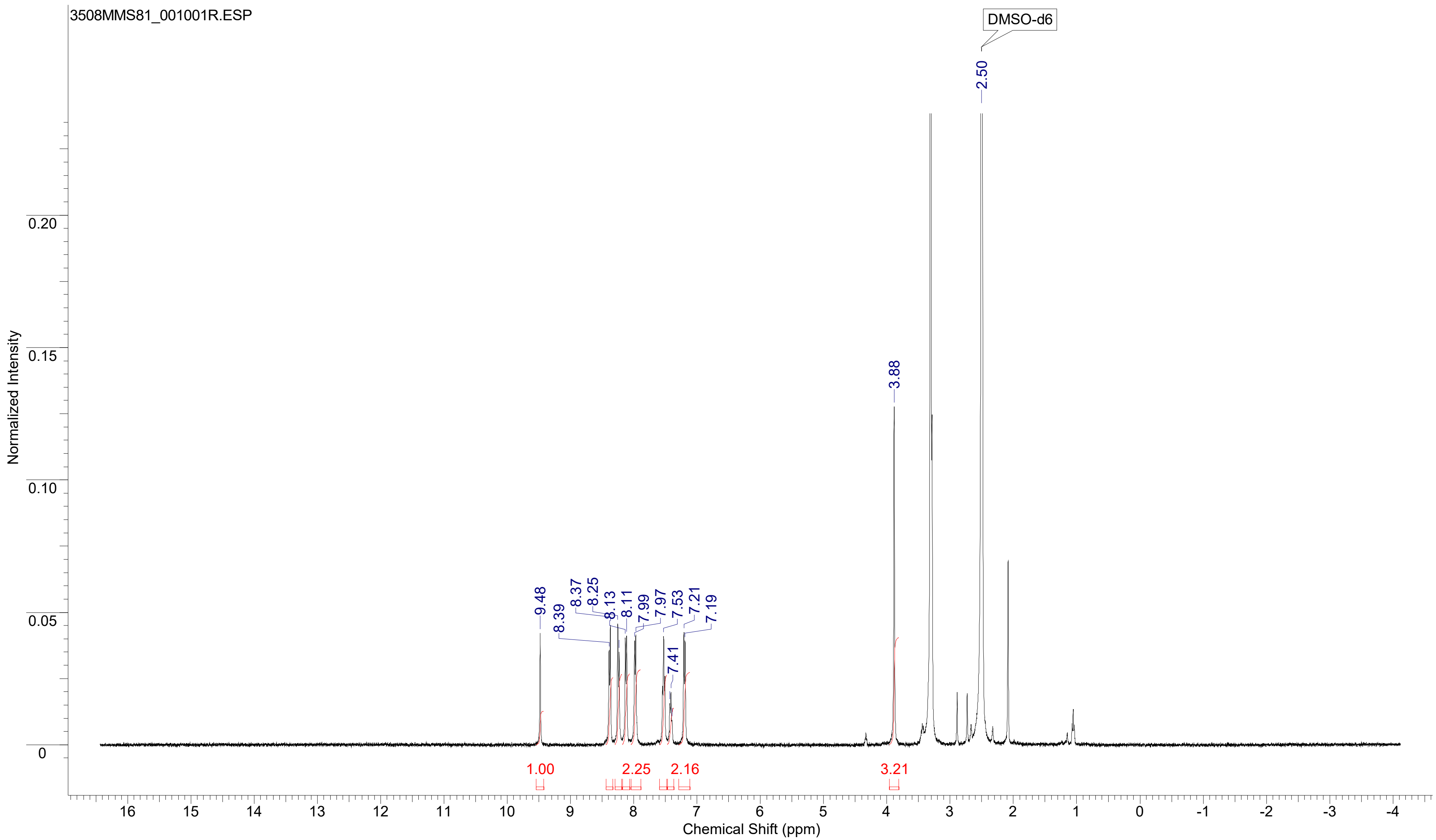

Figure S8. ^{1}H NMR spectrum of compound **3e**.

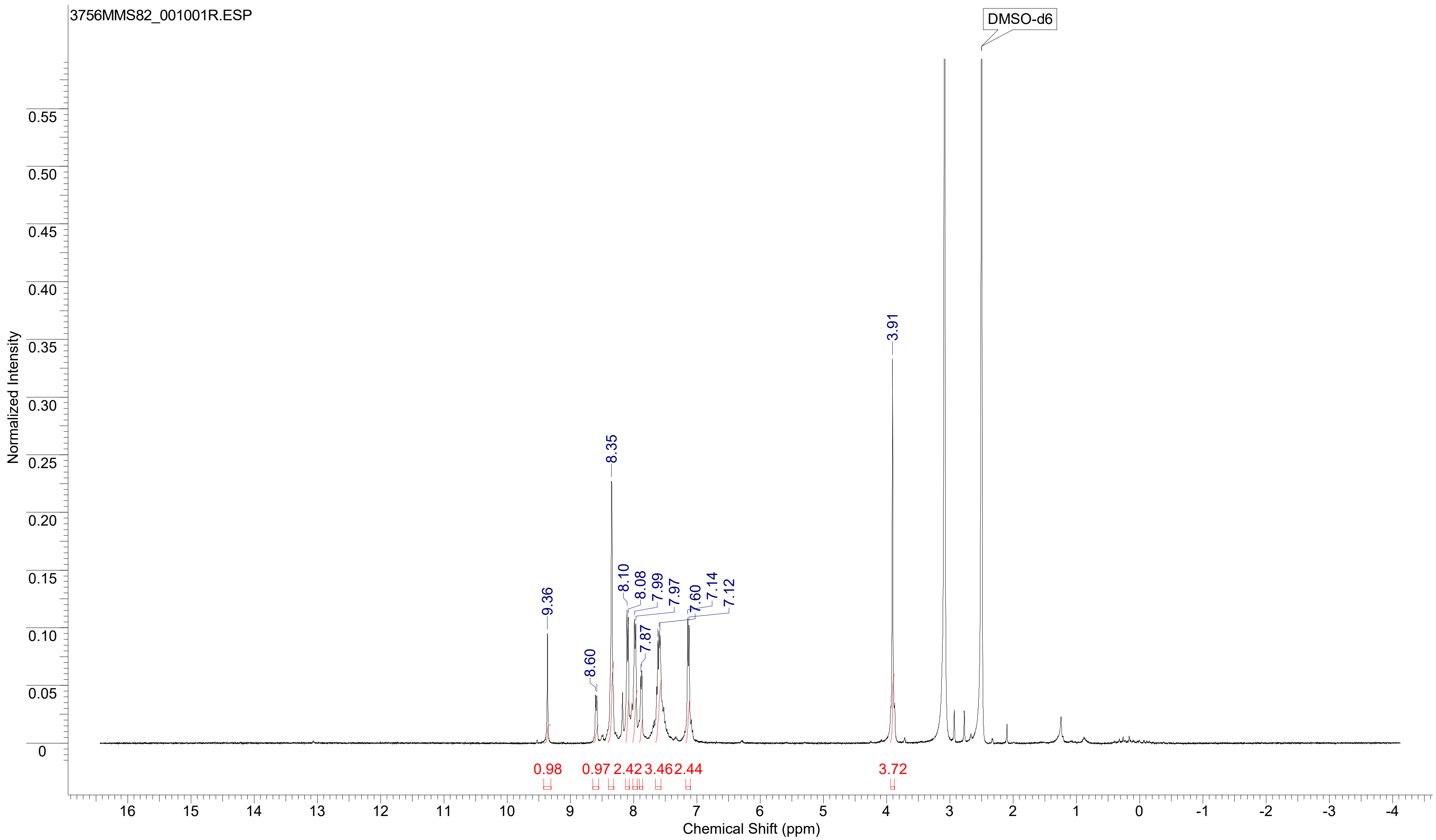

Figure S9. ^{1}H NMR spectrum of compound **3f**.

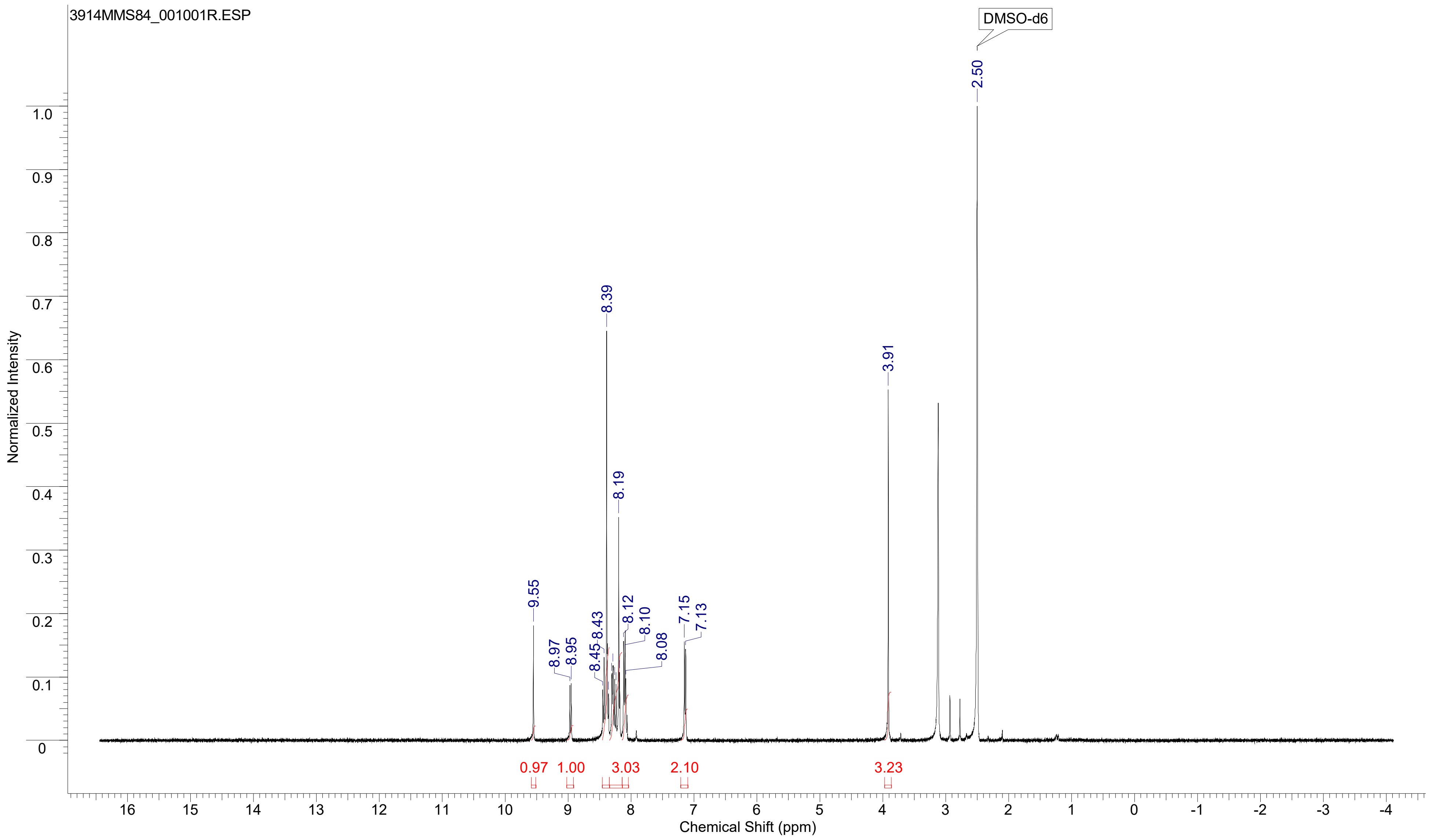

Figure S10. ^{1}H NMR spectrum of compound **3g**.
